# Link between graphene features and the resulting functionality of quasi-van der Waals Zn_3_P_2_

**DOI:** 10.1039/d5ce00351b

**Published:** 2025-10-29

**Authors:** Thomas Hagger, Helena Rabelo Freitas, Chiara Mastropasqua, Ahmed El Alouani, Stefano Marinoni, Nico Kawashima, Raphael Lemerle, Kamil Artur Wodzislawski, Didem Dede, Silvana Botti, Maria Chiara Spadaro, Valerio Piazza, Adrien Michon, Jordi Arbiol, Anna Fontcuberta i Morral

**Affiliations:** a Laboratory of Semiconductor Materials, Institute of Materials, School of Engineering, Ecole Polytechnique Fédérale de Lausanne 1015 Lausanne Switzerland anna.fontcuberta-morral@epfl.ch; b Catalan Institute of Nanoscience and Nanotechnology (ICN2), CSIC and BIST Campus UAB, Bellaterra Barcelona Catalonia 08193 Spain; c Université Côte d'Azur, CNRS, CRHEA Rue Bernard Grégory 06560 Valbonne France; d Research Center Future Energy Materials and Systems of the University Alliance Ruhr and ICAMS, Ruhr University Bochum Universitätsstraße 150 D-44801 Bochum Germany; e Institut für Festkörpertheorie und-Optik, Friedrich-Schiller-Universität Jena Max-Wien-Platz 1 07743 Jena Germany; f Department of Physics and Astronomy “Ettore Majorana”, University of Catania, CNR-IMM via S. Sofia 64 95123 Catania Italy; g ICREA Pg. Lluís Companys 23 08010 Barcelona Catalonia Spain

## Abstract

Zn_3_P_2_, made from earth-abundant elements, is a promising candidate for thin-film solar cells but faces limitations due to difficulties in achieving n-type doping and its large lattice mismatch with commercial substrates and a high thermal expansion coefficient, causing defects and cracks. Graphene substrates can address these challenges thanks to its weak van der Waals interactions with Zn_3_P_2_ allowing for mechanical transfer of the thin film and strain-free growth. This study compares five graphene substrates for quasi-van der Waals epitaxial (q-vdWe) growth of polycrystalline Zn_3_P_2_ thin films using molecular beam epitaxy. Surface features like steps and wrinkles on graphene were identified as main nucleation sites for Zn_3_P_2_, provided the graphene has minimal point defects. The highest-quality thin films, with the largest grain sizes, were grown on H-CVD graphene on the Si-face of 6H-SiC, featuring solely terraces of atomic height. All substrates showed comparable growth windows for crystalline Zn_3_P_2_, with higher growth temperatures improving crystal quality, as indicated by enhanced photoluminescence. Cryo-cathodoluminescence measurements revealed spatially localized sub-bandgap emissions, potentially linked to localized strain fields at grain boundaries of up to ±3% as identified by cross-sectional transmission electron microscopy. This work provides insights into advantages and drawbacks of utilising q-vdWe to produce Zn_3_P_2_ thin films for solar cell applications and highlights the effects of graphene substrate choice and growth parameters on Zn_3_P_2_ film quality.

## Abstract 2

Zn_3_P_2_ growth on graphene: triangular grain nucleation on atomic steps of graphene substrates and its growth window.

## Graphical abstract

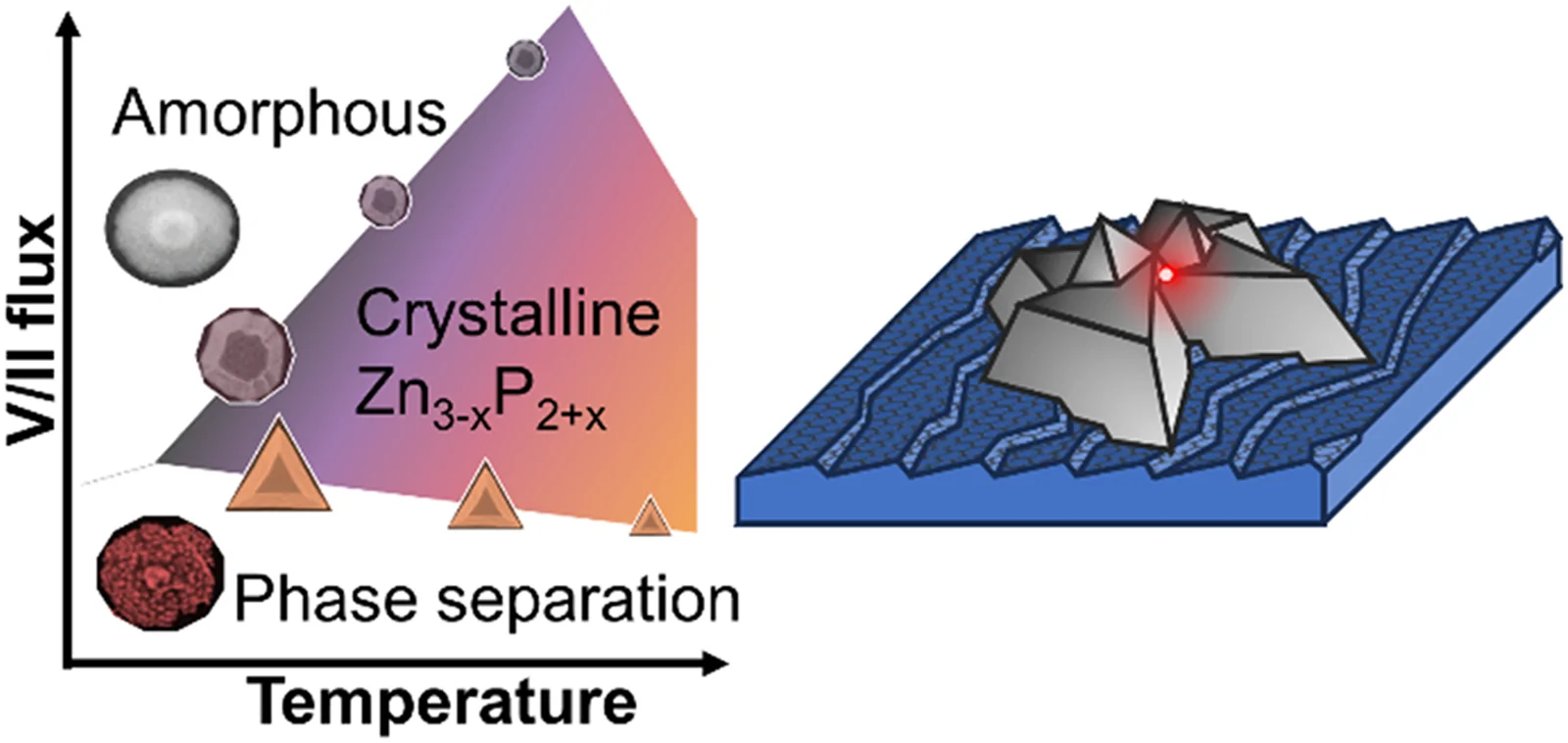

## 1 Introduction

In the pursuit of sustainable and efficient energy solutions, photovoltaics (PVs) have emerged as a helpful technique to reduce CO_2_ emissions in electric power generation. Today, the PV market is dominated by silicon, an earth abundant semiconductor with an indirect bandgap. Achieving peak performance with silicon requires a minimal thickness of PV devices of around 40 μm (ref. 1) and an energy intensive purification step of silicon. Moreover, the conversion efficiency of current silicon technology already approached the thermodynamic limit, leaving little room for performance improvement.^2^ In quest of expanding solar technology worldwide in a short time frame, democratizing PV technology relies on cost reduction by diversifying the active materials. With this in mind, scientists have adopted numerical material discovery platforms to uncover what would be the most optimal material for photovoltaic conversion.^3^ Major criteria are the capability of high absorption of solar radiation and the use of elements that are abundant and easy to extract from the Earth's crust. Other studies go through a similar search effort based on existing experimental work, pointing out reasonable silicon contenders.^4^ Among the materials that attract attention, zinc phosphide (Zn_3_P_2_) constitutes a potential semiconductor absorber for PV applications due to its suitable electronic and optical properties, coupled with the earth abundance of Zn and P.^4–6^ This makes Zn_3_P_2_ an interesting candidate for advancing the frontier of solar energy conversion technologies in a sustainable manner.

Zn_3_P_2_ exhibits a direct bandgap around 1.5 eV, high absorption coefficient (>10^4^ cm^−1^)^7–10^ in the visible range and minority carrier diffusion length of more than 5 μm.^11,12^ Zn_3_P_2_ is intrinsically p-type due to the low formation energy of p-type dopants such as Zn vacancies and P interstitials.^6,13–16^ These defects and self-compensation effects prevent the reliable production of n-type Zn_3_P_2_ and so far only one study reported n-type doping related to excess Zn.^8^ Considering its bandgap, Zn_3_P_2_ would exhibit a maximum theoretical efficiency limit of ∼33%.^17^ The highest conversion efficiency of around 6% was reported in 1981 by Bhushan *et al.* for a Mg–Zn_3_P_2_ based solar cell.^18^ According to initial studies on the material, what limits device efficiency in Zn_3_P_2_ is the need for synthesis resulting in high crystalline quality in a reproducible manner.

The challenge in epitaxial growth is related to its large tetragonal crystal structure (lattice constants *a* = *b* = 8.089 Å, *c* = 11.45 Å)^19^ and thermal expansion coefficient (1.4 × 10^−5^ K^−1^),^20^ matched by none of the available commercial substrates.^21^ In recent years, Zn_3_P_2_ thin films were epitaxially grown on GaAs or InP substrates as they offer the smallest lattice-mismatch especially in the arrangement of the group V elements.^6,22^ The best conversion efficiency for a Zn_3_P_2_/InP heterojunction solar cell was 4.4%, reported by Paul *et al.*^23^ So far, the highest structural and functional quality of Zn_3_P_2_ was obtained *via* selective area epitaxy (SAE).^24,25^ In SAE, growth is initiated in predefined nanoscale areas. The reduction of the contact area with the substrate alleviates the strain, due to the lattice mismatch between the substrate and Zn_3_P_2_, leading to an improvement in crystalline quality. The use of InP as a substrate however is non-desired due to the scarcity of indium. Alternatives to InP as a substrate are highly desired in view of achieving a solution using mostly materials made of earth-abundant elements.

Recently, Paul *et al.*^26^ demonstrated the successful growth of crystalline Zn_3_P_2_ on graphene substrates and provided a promising strategy to circumvent the absence of a lattice-matched earth-abundant commercial substrate. This technique, called quasi-van der Waals epitaxy (q-vdWe), first introduced by Atsushi Koma,^27^ was widely explored and optimised for a wider range of different 2D materials, such as epitaxial layers and substrates.^28,29^ This method makes use of weak van der Waals bonds between the epitaxial film and substrate to achieve an equilibrium crystal structure without the need of an epitaxial relation with the substrate.^30^ While graphene introduces a certain crystallographic order, the lack of covalent bonds with the thin film leads to a relaxation of the lattice matching condition. Consequently, this enables the growth of films with high lattice mismatch relative to the substrate, promoting a defect- and strain-free interface. The drawback of this technique is that it gives rise inherently to polycrystalline thin films precisely due to the weak van der Waals forces.

While the polycrystallinity of the Zn_3_P_2_ thin film might be a setback for the motivation of using q-vdWe, the growth on 2D materials allows mechanical exfoliation, which brings a major advantage compared to the conventional epitaxial growth of Zn_3_P_2_. The possibility of transferring the thin films from the growth substrate on any other platform offers more freedom in device design compared to the epitaxially grown thin films, where the substrate is part of the final device. Transfer processes such as gold-to-gold thermo-compression bonding,^31^ or using thermal release tape not only allow for the creation of flexible devices but also the reuse of the growth substrate.^32^ Selective contact based solar cell devices could therefore be good candidates to circumvent the n-doping issues by optimising device design focusing on p-type Zn_3_P_2_ as an absorber layer.

The achievement of growing Zn_3_P_2_ on commercial polycrystalline graphene highlights the importance of graphene defects and grain boundaries in the growth initiation and progress.^26^ This has also been shown for other materials such as CuZnMn.^33^ Commercially available graphene exists in quite diverse forms, each of them with different topographical features and concentration of defects. Depending on the fabrication process, one can find mono- or poly-crystalline layers, on the micron or wafer scale, defective or very high quality. These features are expected to have a dramatic impact on the use of graphene as an epitaxial substrate. However, to the best of our knowledge, there has been no study that examined the role of the different qualities of graphene substrates in terms of defectiveness and surface topography and their influence on the quasi-van der Waals epitaxial growth and the consequent quality of the epilayer.

Our work aims to compare the nucleation, growth outcome and impact on material properties of Zn_3_P_2_ grown on various graphene substrates of different quality. We used graphene produced on the C-face and Si-face of SiC by annealing, grown by CVD under hydrogen on Si-face SiC, graphene grown by CVD on copper and transferred on SiO_2_, and finally graphite sheets. These five fabrication processes led to different graphene qualities. We highlighted the importance of the graphene substrate topography and structural quality, which will be helpful to identify the right choice of substrate for future projects and applications. We provide a growth window, serving as a guideline to achieve and tune high quality polycrystalline Zn_3_P_2_ films by quasi-van der Waals epitaxy.

## 2 Experimental

### 2.1 Graphene substrate description

Zn_3_P_2_ is grown on five different graphene substrates as summarised in Table 1. The first substrate consists of epitaxial monocrystalline graphene grown on the Si-face of 6H-SiC by CVD under hydrogen.^34^ In this technique, the hydrogen environment prohibits carbon excess on SiC, so that graphene growth requires an external carbon source (propane in this case).^35^ Besides the method allows graphene to grow with different degrees of crystallinity and various interfaces with the substrate.^36^ For this study, graphene was grown under these conditions: 1550 °C and low hydrogen ratio,^37^ leading to the formation of single monocrystalline graphene with typically 5% of bilayer patches. A buffer layer (BL) made of sp^2^ and sp^3^ carbon atoms is present at the graphene/SiC interface, similarly to what can be obtained using Si sublimation. The substrate used here, with an average miscut of around 0.07°, leads to the formation of terraces with heights ranging between 0.25 and 1.5 nm and a maximum width of 400 nm. We refer to this substrate as the Si-face H-CVD substrate.

**Table 1:** Summary of the five graphene substrates used in this study

Substrate name	Wet-transferred	Si-face H-CVD	C-face annealed	Si-face annealed	Graphite
Substrate description	SiO_2_/Si	6H-SiC	4H-SiC	4H-SiC	Graphite
Graphene crystallinity	Poly	Mono	Poly	Mono	Poly
Growth method	CVD	CVD	Annealing	Annealing	Exfoliation
Topography	Wrinkles	Atomic steps	Terraces	Terraces	Wrinkles
	Particles		Atomic steps	Atomic steps	Particles
			Wrinkles	Wrinkles	
			Particles	Particles	
Defects	Grain boundaries	Point defects	Grain boundaries	Graphene edges	Graphene edges
	Graphene edges		Graphene edges	Point defects	Point defects
	Point defects		Point defects		
Graphene coverage	100%	100%	∼50%	100%	100%
Bi-/Multilayers present	Yes	∼5% + BL	Yes	∼18–25% + BL	Yes
Stable during growth	No	Yes	Yes	Yes	Partially
Zn_3_P_2_ delamination	Yes + loss of graphene	Yes	Yes	Yes	No
Substrate provider	Graphenea	CNRS-CRHEA^36^	Graphensic	Graphensic	Nanografi

The second substrate is covered by epitaxial monocrystalline graphene grown on the Si-face of 4H-SiC purchased from Graphensic, which was produced through Si sublimation. In this case the substrate turns to be covered by monolayer graphene mixed with a small amount of bi-layer graphene. Similar to the first substrate, the Si sublimation leads to the formation of a BL below the graphene. The surface contains terraces of height up to 10 nm containing smaller steps and particles. Wrinkles in the graphene can be found at the edge of the terraces. This substrate is referred to as the Si-face annealed substrate.

The third substrate consists of commercially available epitaxial polycrystalline graphene, produced on the C-face of 4H-SiC by high temperature annealing under argon. The annealing leads to a partial coverage of the substrate with monolayer and also bi-/multilayer graphene islands. The surface of these substrates exhibits large steps of up to 10 nm height, much larger than that of the Si-face H-CVD substrate, and the graphene formed very prominent wrinkles. We refer to this substrate as the C-face annealed substrate.^38–40^

The fourth substrate corresponds to commercially available polycrystalline graphene, which is grown by CVD on copper foil and transferred on a SiO_2_/Si substrate with a wet transfer method. This substrate contains features such as wrinkles, particles below the graphene, grain boundaries and small patches of bi-/multilayer graphene, which provide edges and dangling bonds. In this study we refer to this substrate as the wet-transferred substrate.^5,41–43^

The fifth substrate is a graphite sheet of around 35 μm thickness. The surface exhibits large height variations up to 1 μm in addition to cracks, large folds and wrinkles due to the overlap of graphene layers. The graphene layers provide grain boundaries, which tend to accumulate close to each other, and in turn leave certain areas smooth and free of such features. We refer to this substrate as the graphite substrate.^44,45^

All but the first substrate are commercially available. The Si- and C-face annealed substrates were purchased from Graphensic, wet-transferred substrates were purchased from Graphenea, and graphite substrates were purchased from Nanografi.

### 2.2 Zn_3_P_2_ growth procedure

Zn_3_P_2_ was grown in a Veeco GENxplor Molecular Beam Epitaxy (MBE) system. The Zn is provided by a valved cracker cell and the P_2_ from a valved GaP cell from MBE-Komponenten with an integrated Ga capturing system. The V/II (P_2_/Zn) flux ratios are obtained from measurements of the fluxes with a beam flux monitor, directly after growth. The indicated temperatures refer to nominal temperatures, measured by a thermocouple above the manipulator. Before growth, all substrates were annealed in an ultra-high vacuum (UHV) below 5.0 × 10^−10^ torr at 650 °C for 2 h to desorb water and other airborne molecules from the surface. This was conducted in a chamber adjacent to the growth module. Subsequently, 1 h annealing was carried out at the same temperature in the growth chamber. After annealing, the substrate was cooled down to the growth temperature and exposed to P_2_ for 5 min, followed by the growth of Zn_3_P_2_. The values for the beam equivalent pressures (BEP) of Zn were: wet-transferred = 8.0 × 10^−7^ torr while for the other substrates 1.45 × 10^−6^ torr. The P_2_ flux was adjusted to vary the V/II (P_2_/Zn) flux ratios. The wet-transferred substrates used lower fluxes due to a much higher growth rate, which allowed for better control of the growth outcome and the prevention of full delamination of the Zn_3_P_2_ film, which will be discussed in section 3.3.

### 2.3 Measurement techniques

Scanning electron microscopy (SEM) images were acquired using a Zeiss Merlin FE-SEM with a Gemini column. The images were taken with an in-lens secondary electron detector at 2 keV accelerating voltage and a current of 80 pA.

Atomic force microscopy (AFM) imaging was conducted with a Bruker Fastscan AFM operated in tapping mode. The tip used was a Bruker Fluidscan+ tip.

X-ray diffraction spectroscopy (XRD) measurements were taken in the *θ*–*θ* configuration with a Panalytical Empyrean XRD thin film diffractometer with a Cu (K-α) X-ray source of 1.54 Å.

Photoluminescence spectroscopy (PL) was performed at room temperature (RT) with a Renishaw inVia with a 532 nm laser and 100× objective (0.85 NA), resulting in an illumination intensity of ∼393 μW μm^−2^. Single point measurements were taken for 1 s averaged over 60 acquisitions while maps used 1 acquisition per pixel. The laser power used for all measurements was 180 μW. The grating used was 300 lines per millimetre.

Raman spectroscopy was performed at room temperature with a Renishaw inVia with a 532 nm laser and 100× objective (0.85 NA). Single point measurements were taken for 5 s averaged over 60 acquisitions, while maps and line-scans were taken for 1 acquisition per pixel. The laser power used for all measurements was around 180 μW and the grating used was 1800 lines per millimetre.

Cathodoluminescence spectroscopy (CL) was performed at 10 K with an Attolight Allalin setup. All acquisitions were conducted with an Andor Newton DU920P-BEX2-DD Si-CCD, an accelerating voltage of 5 keV and a current of 0.89 nA and an acquisition time of 2 s per pixel. The grating used had 150 lines per millimeter and a blaze wavelength of 500 nm. The data treatment on the CL spectra was a background subtraction of the dark count of the CCD and a Savitzky–Golay low-pass filter (parameters: window size = 5 pixel, polynomial order = 3 and derivative = 0) was applied due to the high noise in the signal.

Electron transparent transmission electron microscopy (TEM) samples were prepared using focused ion beam (FIB) processing with a Thermo Fisher FIB Helios 5 UX or HELIOS 600 FIB system. Bright field TEM imaging was performed with an FEI Talos operating at 200 kV. HRTEM (high resolution transmission electron microscopy) imaging was performed using a field emission gun FEI Tecnai F20 microscope operated at 200 kV. STEM HAADF (high-angle annular dark field scanning transmission electron microscopy) images and STEM EDS (energy dispersive X-ray spectroscopy) maps were acquired using a double corrected Thermo Fisher Spectra 300 microscope operated at 300 kV. Geometrical phase analysis (GPA) was performed on experimental images using the licenced GPA plug-in available in Gatan Digital Micrograph.

## 3 Results and discussion

### 3.1 Growth on different graphene substrate qualities

**Fig. 1:**
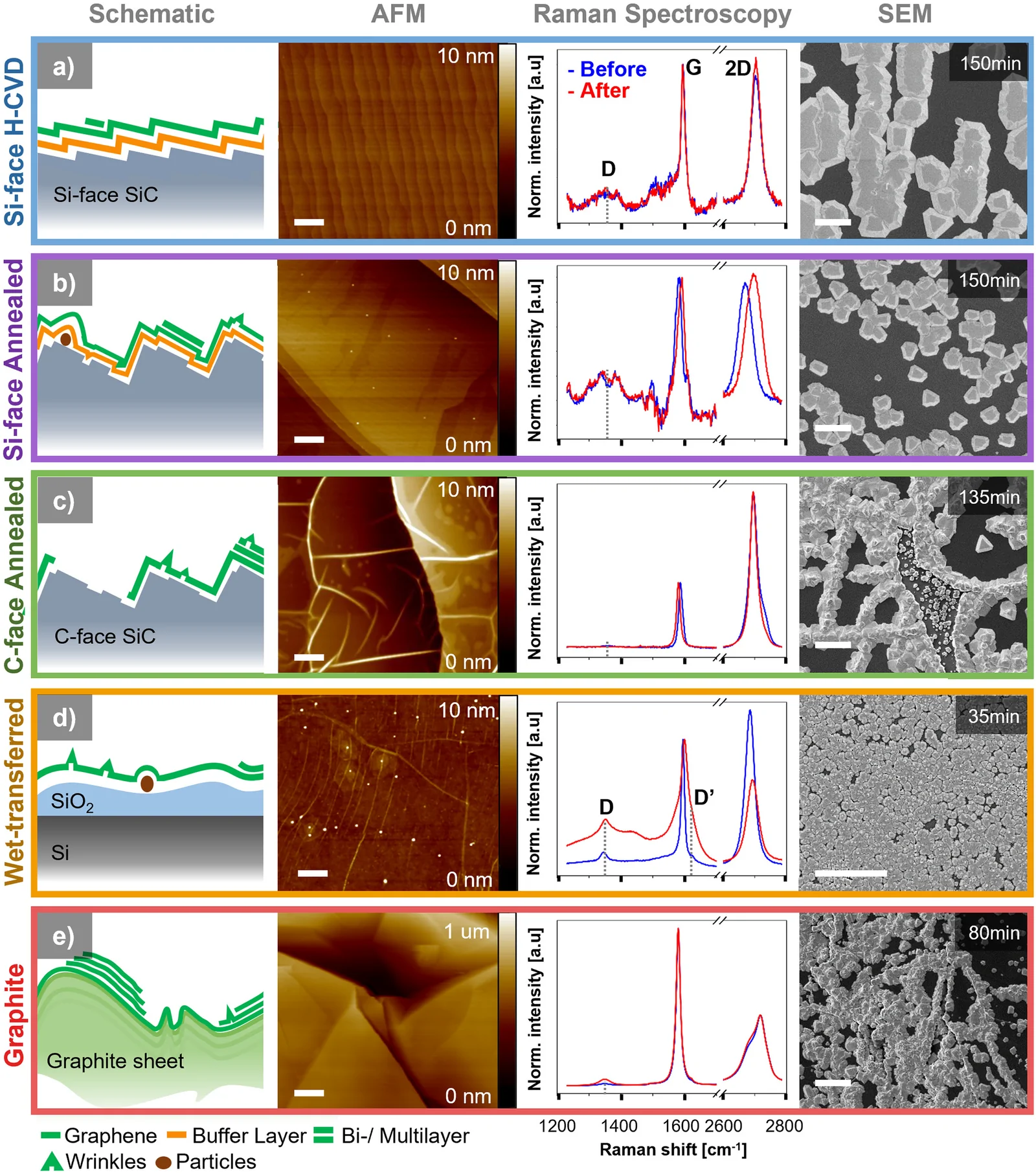
Summary of the topography, graphene quality and growth outcome of Zn_3_P_2_ on the five discussed graphene substrates. (a) Si-face H-CVD; (b) Si-face annealed; (c) C-face annealed; (d) wet-transferred; (e) graphite. Column one shows the schematic representations of all substrates, indicating topographical features present and the quantity of graphene layers. Column two presents the AFM images of the corresponding substrate after a growth process without material fluxes. The colour scale indicated on the right is the same for (a–d). Raman spectra of all graphene substrates are shown in column three, normalised to the G peak. Blue spectra were acquired before growth and the red ones after growth. Additional spectra of different positions are given in Fig. S1 in the SI. Column four shows the SEM images of the grown Zn_3_P_2_ on each substrate under the same growth conditions (200 °C, V/II flux = 1) with different growth times adapted to the substrate, which are indicated in min in the top right. Scale bars of AFM images are 1 μm and 2 μm for SEM images.

In this section, we compare the growth outcome of Zn_3_P_2_ on the five graphene substrates. The schematics in Fig. 1 summarise the presence and absence of multilayer graphene and the topography of each graphene substrate. The topography is also shown in the AFM images taken after a growth process without the use of material fluxes. A large variation of the topography can be observed among different substrates. The Si-face H-CVD substrate shows only atomic steps while all others contain a variety of features such as terraces, wrinkles and particles. The quality of our substrates was assessed with Raman spectroscopy. The spectra given are single point measurements, acquired before (blue) and after (red) growth to evaluate damage during the growth process. Additional Raman spectra of different locations are provided in Fig. S1 in the SI. A typical Raman spectrum of graphene is composed of three main peaks. A defect-related peak called the D peak around 1350 cm^−1^, a fundamental G peak around 1600 cm^−1^, and a second order peak called 2D around 2690 cm^−1^.^46^ In the case of very defective graphene a second defect related peak called D′ around 1625 cm^−1^ appears or broadens the G peak.^46^ Studies have shown that the *I*_D_/*I*_G_ peak intensity ratio relates to the defectiveness of graphene while the *I*_2D_/*I*_G_ ratio and WHM_2D_ give information about the quantity of graphene layers stacked on top of each other. Typically, the *I*_2D_/*I*_G_ ratio decreases and the FWHM_2D_ broadens with an increasing number of graphene layers. The ratio and the 2D peak shape also depend on the alignment of the multilayer stack and excitation wavelength.^47–54^

The growth outcome of Zn_3_P_2_ on each of the substrates is evaluated using the top view SEM microscopy image under the same growth conditions (200 °C, V/II flux ratio of 1). These growth conditions were selected due to the reasonable growth time and achievable flux ranges of the cells. Under the indicated growth conditions, we observed that the grain morphology on all graphene substrates resembles the one reported by Paul *et al.*^26^ The growth exhibits a pyramidal grain shape due to the 〈111〉_*C*_ growth direction in the quasi-cubic system of Zn_3_P_2_ and has a rotational degree of freedom around the growth axis, resulting in a polycrystalline film. The rotational degree of freedom is due to the weak van der Waals forces between graphene and Zn_3_P_2_,hindering lattice matching as in classical epitaxy. Changes of the grain morphology were observed for different growth conditions and will be discussed in section 3.2. The Zn_3_P_2_ follows the Volmer–Weber growth, typical for materials grown by quasi-van der Waals epitaxy, due to the low sticking coefficient of adatoms on graphene compared to the islands.^29,55,56^

Looking at the growth outcome of Zn_3_P_2_ on the Si-face H-CVD substrate in the SEM image in Fig. 1a, one can see that the grains tend to nucleate on the steps. When comparing the distance between steps to that of the grains, it can be observed that not every atomic step exhibits nucleation. The Raman spectra of the substrate shows no clear presence of the defect peak around 1350 cm^−1^ and the graphene is stable during the growth process, as Raman spectra before and after growth are similar. Based on these measurements and observations we conclude that the topographical features dominate in the nucleation process. Similar behaviours are observed for the growth on the Si-face and C-face annealed substrates, shown in Fig. 1b and c. Here the nucleation predominantly occurs on topographical features such as the wrinkles, particles and steps while Raman spectroscopy reveals low defectiveness (small D peak) and stable graphene. In the case of the Si-face annealed substrate, one can see an increased nucleation density on the terraces compared to the growth on the Si-face H-CVD substrate. This is likely due to the presence of smaller steps and particles, as seen in the AFM image in Fig. 1b. It has to be noted that the Raman spectrum of the graphene overlaps with SiC modes. The SiC background was subtracted as shown in Fig. 1 for the Si-face H-CVD, Si-face and C-face annealed substrates and we assume the broad peak around 1350 cm^−1^ to originate from the data processing rather than the defectiveness of the graphene. The original data are provided in Fig. S1 in the SI.

Based on these observations, we interpret that the effect of topographical features on the growth outcome is similar to what is reported in classical epitaxy,^57^*i.e.* abrupt height changes (steps, wrinkles, particles) lead to higher nucleation density due to increased adatom density and lower surface energy, which traps adatoms and facilitates stable island formation compared to flat substrates.^57–61^

A different growth behaviour is observed on the wet-transferred graphene. Despite having topographical features (particles, wrinkles), the nucleation occurs more homogeneously and very densely over the whole substrate leading to much smaller grains. We attribute this change to a lower quality of the graphene substrates (high density of point defects) visible through the very pronounced and broad D peak in the Raman spectra of the wet-transferred substrate. Based on these observations, we expect the nucleation to occur on defects in the wet-transferred graphene. It is still unclear of what nature these defects are and why their density increases during the growth process in wet-transferred substrates, while all SiC substrates (Si-face H-CVD, C-face, Si-face) are stable. We assume that defects created in the graphene layer during the transfer process make the substrate unstable for our growth process.

The graphite substrate shows two different properties. Smooth regions, which are covered with a high quality layer of graphene, do not show the presence of a D peak and remain unchanged during growth. As graphene patches in graphite are limited in size, there are inevitably grain boundaries of these graphene patches, which provide dangling bonds. Grain boundaries were observed to accumulate close to each other, at folds and wrinkles in the substrate. In these regions, Raman spectra also show a D peak which also increases in intensity during the growth process. Depending on the location and presence of the D peak, graphite seems to have a topography or defect driven nucleation process. Refer to Fig. S1 for additional Raman spectra and Fig. S2 in the SI for SEM micrographs of the graphite surface and growth homogeneity.

DFT calculations have shown that the sticking coefficient (binding energy) for adatoms increases as more graphene layers are stacked on top of each other.^62^ This increase in adsorption could be translated into an increase in growth rate on multilayer graphene compared to single-layer. The C-face annealed graphene substrate provides entire terraces covered in different graphene thicknesses, as visible in the Raman maps provided in Fig. S3 in the SI.

Additionally a significant variation in growth rate is observed on different terraces but does not seem to be correlated to the number of graphene layers based on these preliminary results. We suspect that any effect due to the graphene layer number is overshadowed by topographical variations of the substrate surface.

To summarise, the choice of graphene substrate has no influence on the Zn_3_P_2_ grain morphology and therefore the grain morphology is independent of the quality and topography of the graphene substrate. Nucleation density and location, on the other hand, are highly dependent on the choice of substrate. In the case where the graphene exhibits high quality (low intensity in the Raman D peak), the nucleation is topography driven, with nucleation occurring mainly on topographical features. A high defectiveness of the graphene will lead to nucleation predominantly on defects such as point defects or dangling bonds and significantly increases the nucleation density. A higher nucleation density, leading to more grain boundaries, is less preferable for a solar cell absorber, as grain boundaries could have a significant impact on the optical and electronical properties of the material. With this in mind, the Si-face H-CVD substrate is the best candidate amongst the investigated graphene substrates to produce thin films for solar applications. Therefore further explorations will be performed only on epitaxial growth of Zn_3_P_2_ only on Si-face H-CVD substrates.

### 3.2 Growth window for Zn_3_P_2_

We now move to the investigation of the growth window, *i.e.*, the parameter range enabling growth of crystalline Zn_3_P_2_ and how changes within this window affect the material.

**Fig. 2:**
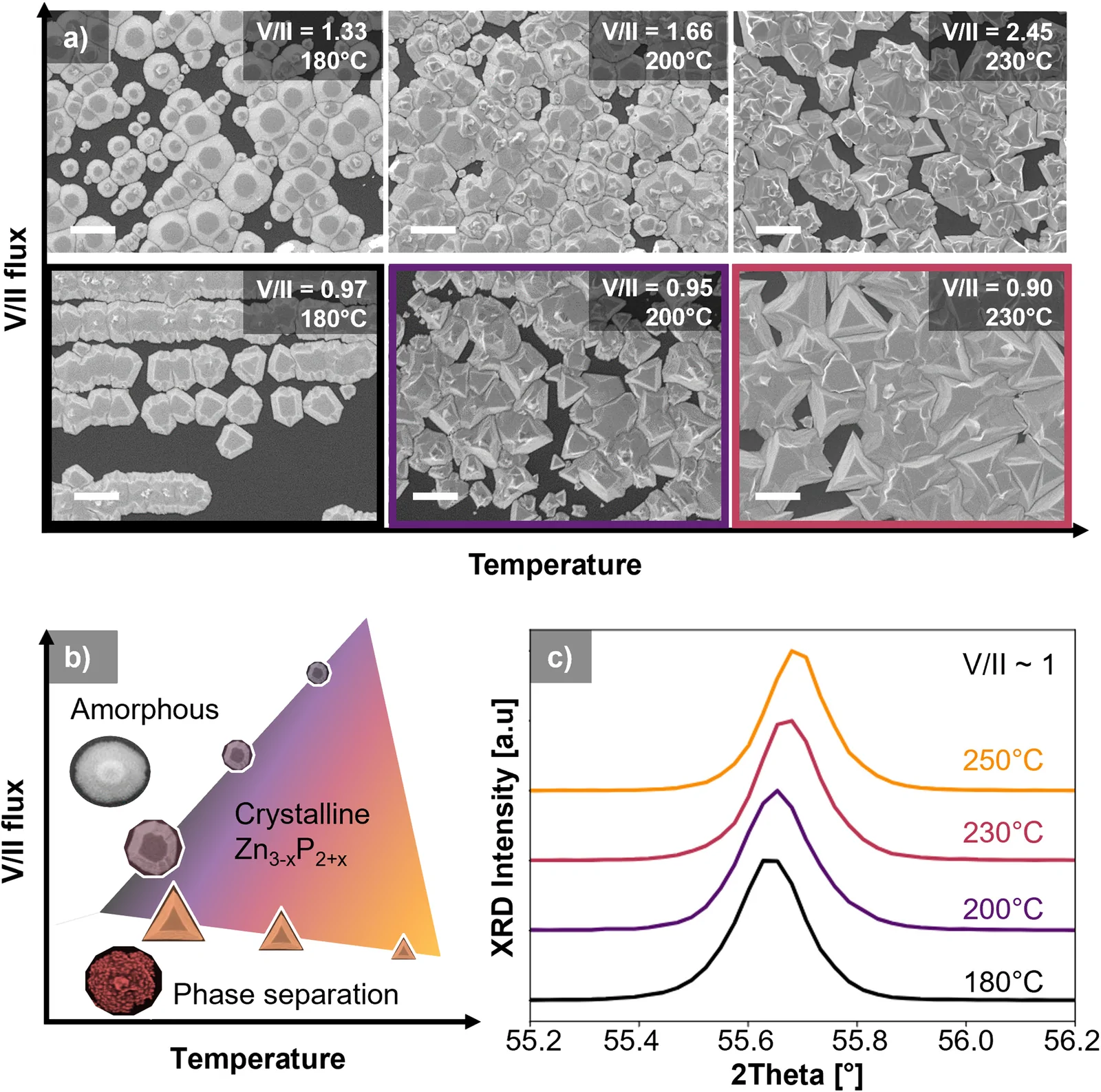
(a) SEM images of crystalline Zn_3_P_2_ (Zn_3−*x*_P_2+*x*_) grown on Si-face H-CVD substrates for different growth temperatures and V/II flux ratios. Scale bar corresponds to 1 μm; (b) a schematic representation of the Zn_3_P_2_ growth-window of any graphene substrate. Smaller nuclei indicate the decrease in growth rate; (c) the 404 peak of Zn_3_P_2_ in an XRD Bragg–Brentano (*θ*–*θ*) configuration grown at four different growth temperatures with V/II flux ratios ≈ 1. The colours indicate the growth conditions in (a).

Fig. 2a shows the top view SEM images of different growth conditions leading to polycrystalline α-Zn_3_P_2_ grown on the Si-face H-CVD substrate, which ensures homogeneous growth over a full chip and produces the largest average grain size among the tested substrates. It has to be noted that the same morphological trend shown in Fig. 2a can be reproduced on the other types of graphene substrates investigated by adjusting the temperature range (SEM micrographs are given in Fig. S5 and S6 in the SI). This observation reinforces the conclusion that substrate quality and growth parameters have independent impact on the thin film quality.

A schematic of the growth window of Zn_3_P_2_ is depicted in Fig. 2b. Outside the growth window of crystalline Zn_3_P_2_, we obtain an amorphous structure for high P_2_ fluxes and zinc segregation for high Zn fluxes. The amorphous growth resembles round balls and the zinc segregation consists of a mixture of Zn_3_P_2_ with Zn clusters. These results are consistent with the findings of Paul *et al.*^26^

Within the growth window, where crystalline Zn_3_P_2_ can be achieved, we have observed a continuous change in grain morphology. Round grains, with a flat top, are obtained for low growth temperatures and/or high V/II flux ratios, and go towards triangular grains for high growth temperatures or low V/II fluxes. STEM-EDS measurements, given in Fig. S4 in the SI, show that this morphology change is accompanied by a change in composition. We detected an increasing zinc content for higher growth temperatures at similar V/II flux ratios. It should be noted that the absolute values of the composition are not accurate due to the difficulty in detecting phosphorus and the lack of a reference on the measurement that would allow calibration.

The change in morphology and composition suggests a higher desorption rate for P_2_ compared to Zn adatoms in the temperature between 180 °C and 250 °C. At temperatures above 250 °C, Bosco *et al.*^6^ observed a drastic decrease in the growth rate of Zn_3_P_2_ associated with a decrease in the Zn sticking coefficient.

In addition to STEM-EDS, XRD measurements of different growth conditions were performed. Fig. 2c shows the 404 peak of Zn_3_P_2_ in the Bragg–Brentano scan of almost fully covered films grown at temperatures ranging from 180 °C to 250 °C at similar fluxes. A wider 2*θ* range of the Bragg–Brentano scans are given in Fig. S9 in the SI. For all Zn_3_P_2_ thin films, only the {101} family of planes is present, indicating that the growth direction introduced by graphene remains unchanged within the growth window. The peak position for different growth temperatures increases in 2*θ* with increasing growth temperature. Growths from 180 °C to 250 °C have peaks at 55.64°, 55.65°, 55.67° and 55.69° leading to a 0.01° increase per 20 °C in growth temperature. Lattice constant variation can only be approximated from these measurements due to the absence of diffraction peaks of other planes compared to the {101} family. Under the assumption that the lattice remains quasi-cubic 
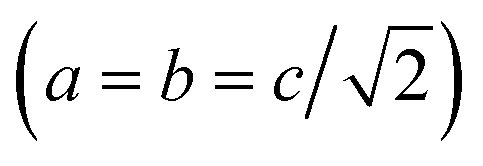
 the lattice contracts by 0.08% from the film grown at 180 °C to the one grown at 250 °C. The magnitude of the observed shift is in agreement with a study performed on thin films of Zn_3_P_2_ grown on InP, which have successfully been correlated to a change in composition with lower 2*θ* values for higher P_2_ content in the film.^15,63^ Bragg–Brentano scans of different growth durations were measured to exclude any XRD peak shift induced by the film thickness and are provided in Fig. S9 in the SI. XRD rocking-curve measurements, given in Fig. S10 in the SI, also indicate a better thin film quality for samples grown at higher temperature. XRD measurements of thin films grown at the same temperature but different V/II flux ratios do not point to a clear trend in the change of crystal quality (more information is given in Fig. S10).

To conclude, here we described the growth window of Zn_3_P_2_ grown on Si-face H-CVD substrates. Growth temperature and V/II flux are found to be correlated to a continuous change in morphology of the grains and in composition. This growth window is consistent over all tested graphene substrates. The observed variation in composition perfectly matches the reports on Zn_3_P_2_ grown on conventional substrates. Section 3.4 will discuss the implications of the different growth conditions on optical properties.

### 3.3 Thin film growth

**Fig. 3:**
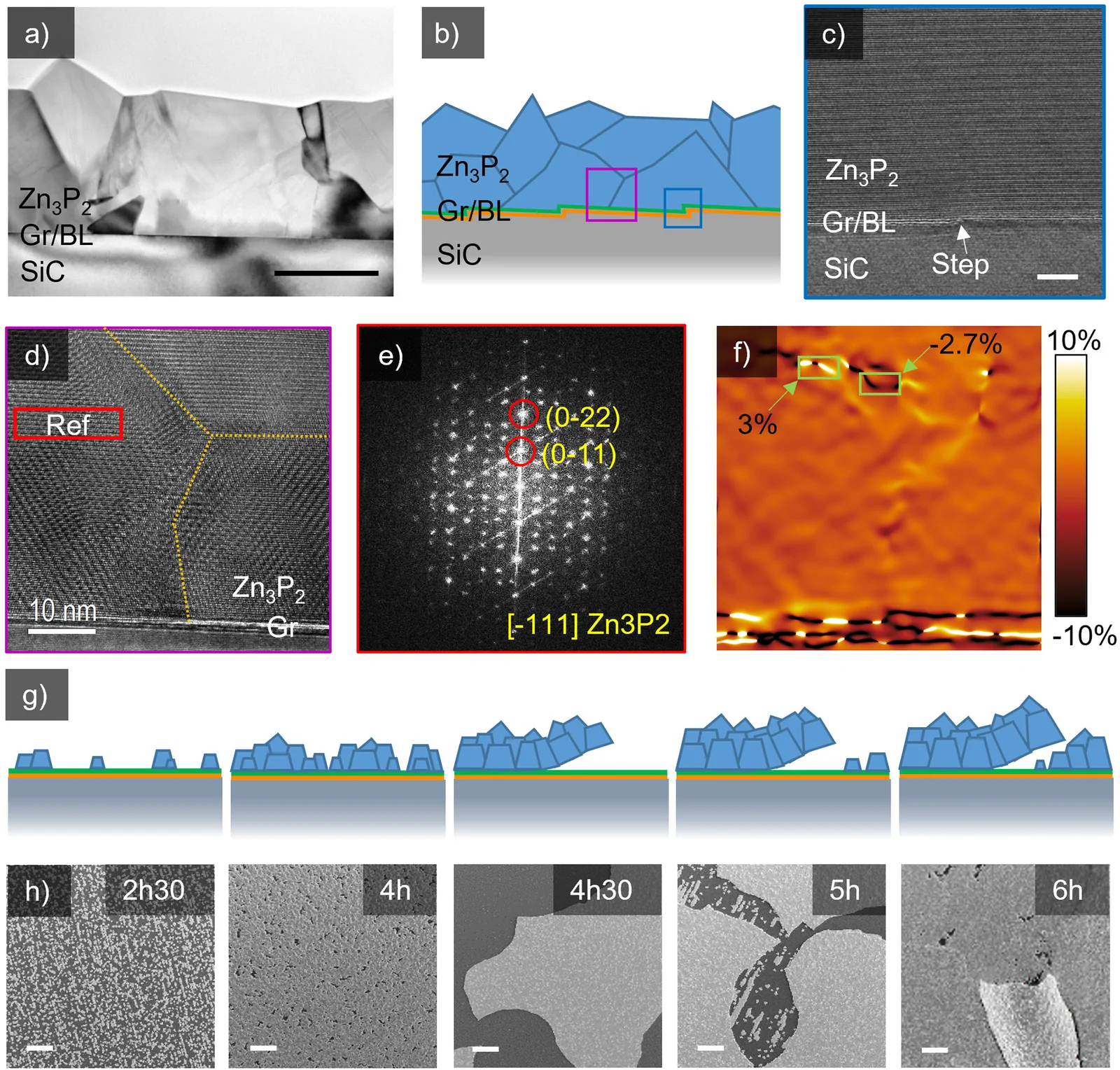
(a) Bright field TEM image of a typical cross-section of a crystalline Zn_3_P_2_ film, here grown on a Si-face H-CVD substrate. The scale bar is 500 nm; (b) schematic representation of a Zn_3_P_2_ film; (c) HRTEM of a grain growing on top of an atomic step as indicated in the schematic. No defect is introduced by the step. The scale bar is 5 nm; (d) HRTEM of multiple grains merging, similar to the purple marked section in the schematic. The scale bar is 10 nm; (e) FFT of the ref. area used in the GPA analysis and marked in red the diffraction spots used for the GPA rotation map given in Fig. S14 in the SI; (f) GPA dilatation map showing localised strain up to 3% at grain boundaries; (g) schematic of the thin film growth, delamination and regrowth process; (h) SEM top view images matching the stage of the schematic for growth durations between 2 h 30 min and 6 h. The growth conditions used were 230 °C and V/II ≈ 1. The scale bar is 10 μm.

The quality of fully grown thin films is key to producing large scale devices. In this section, we discuss the cross section of thin films and present a growth time series, clarifying how the thin film evolves. The bright field TEM image of a cross-section of a film grown at 230 °C and a V/II flux of 1 on a Si-face H-CVD substrate is shown in Fig. 3a. Fig. 3b depicts the schematics of the grain boundaries that one can deduce from the TEM micrograph. The polycrystalline film is composed of grains oriented along the (101) growth plane and randomly rotated around the growth axis perpendicular to the graphene plane. The grains also nucleate on top of Zn_3_P_2_, creating additional grain boundaries within the film.

A closer look at the interface between graphene and Zn_3_P_2_, given in Fig. 3c, showed no defects in the Zn_3_P_2_–graphene interface nor the step edge. This absence of defects, even at the step, suggests that the relaxed lattice-matching conditions at the graphene interface support strain and defect-free growth.

The thin films however contain a significant amount of complex grain boundaries, due to the existence of grains of different orientations merging and competing for growth space. Fig. 3d shows the HRTEM of a section containing multiple grains merged together. A strain analysis was performed with ref. area marked in red. Its fast Fourier transformation (FFT) is given in Fig. 3e with zone axis [−111] and the growth direction along the (0−11) plane. Elongations of the FFT pattern are assumed to be related to the influence of other grains close to the reference area. Fig. S14 in the SI shows the FFT of the other grains present and overlapping within the thickness of the lamella within this HRTEM image, all of the different zone axes, and therefore different rotations. Fig. 3f shows the corresponding dilatation map, with an indication on a localised strain field at a grain boundary calculated for the planes perpendicular to the growth direction that goes up to ±3%. This strain and the grain boundaries can be situated within the thickness of the lamella, as multiple grains are overlapping with each other. The high strain shown in the substrate is an artifact related to the substrate not being in the zone axis, which is due to the missing lattice matching between the SiC and Zn_3_P_2_.

In the frame of classical epitaxy, several studies have shown that coalescence of grains leads to a strain buildup.^64–66^ Due to the randomly rotated grains of our system, it is not surprising to find such high strain between grains as they compete with each other when expanding, even in the absence of covalent bonds between the substrate and epilayer. A recent study has characterised specific, defect-free interfaces in Zn_3_P_2_, but the potential impact of common, dangling-bond-rich grain boundaries and strain on the functional properties remains unclear.^25^

A growth time series is schematically depicted in Fig. 3g and top view SEM micrographs are given in Fig. 3h. The films are grown on Si-face H-CVD substrates at 230 °C and a V/II flux of 1. In the initial stage of growth, grains nucleate continuously on steps, and expand until a full film of various grain sizes is formed. Under the chosen growth conditions this occurs between 4 h and 4 h 30 min. Shortly after achieving full coverage, delamination and cracking of the thin film of Zn_3_P_2_ occur. The film thickness before delamination is close to 1 μm. After delamination, growth resumes identical to the initial nucleation at the exposed steps of the Si-face H-CVD substrate. This indicates that the graphene remains on the substrate after delamination, without significant degradation of the graphene quality. This is supported by the characterisation of the substrate by Raman spectroscopy. Raman spectra of the graphene after delamination are identical to the ones before growth, similarly also for C-face and Si-face annealed substrates. More details are given in Fig. S7 in the SI. Thin films obtained on wet-transferred substrates delaminate at much thinner thicknesses, around 100 nm. We believe this is due to the higher nucleation density, which leads to a higher density of grain boundaries and thus to accumulated strain combined with a faster surface coverage. Interestingly, we observed that in the case of the wet-transferred substrate, the graphene delaminates together with the Zn_3_P_2_ film. This could be explained by a higher density of covalent bonds formed, due to the high density of point defects in the graphene, as indicated by the intense D peak in section 3.1. Delamination has not been observed on the graphite substrate, which could be related to the flexibility of the graphite substrate, its extreme height variations or a very high number of covalent bonds between graphite and Zn_3_P_2_. A detailed investigation of this effect is outside the scope of this article. SEM images of delamination on the different substrates are given in Fig. S10 in the SI.

While this delamination of Zn_3_P_2_ has its drawbacks, it shows the potential that the thin films on graphene can be exfoliated to other substrates, which could allow the reuse of the substrates or the fabrication of flexible devices. Two exemplary transfer methods are described in the SI (Fig. S8).

In summary, TEM data showed localised strain at grain boundaries in fully formed thin films. Additionally, the defect-free interface between Zn_3_P_2_ and the substrate demonstrates the unconstrained growth of quasi-van der Waals epitaxy. From the five tested substrates, only the Si-face H-CVD, Si-face annealed and graphite substrate can be used to grow fully covered films of sufficient thickness for a solar cell absorber layer. The delamination of thin films limits the achievable film thickness, which is related to the nucleation density and graphene substrate quality. Exfoliation can be achieved with various techniques due to the weak van der Waals forces between graphene and Zn_3_P_2_.

### 3.4 Optical characterisation

In this section, we correlate the microstructure with the optical properties to outline the functional quality of the thin films and their potential for application in future devices.

**Fig. 4:**
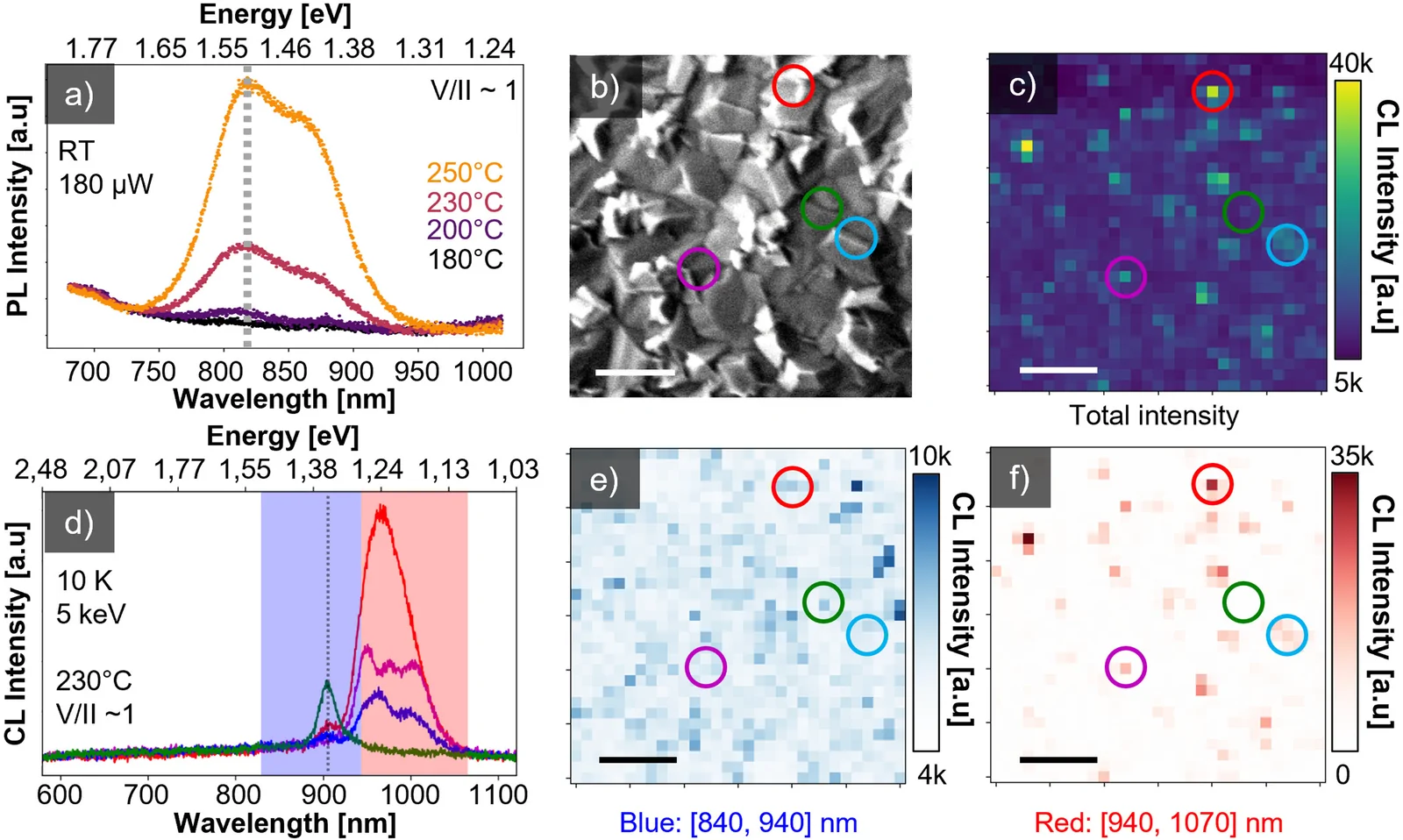
(a) Room temperature PL spectra of four Zn_3_P_2_ films grown at 180 °C, 200 °C, 230 °C and 250 °C with a V/II flux ratio = 1. The films correspond to the same ones measured with XRD in Fig. 2b with the same colour scheme. Dashed line acts as a guide for the eye. (b) Top view SEM image of the region where the CL map was acquired for a Zn_3_P_2_ film grown at 230 °C and V/II flux ratio = 1 for 6 h at 10 K. (c) Panchromatic CL intensity of the 32 × 32 pixel map. (d) Four exemplary CL spectra of the CL map. The positions of the four spectra are marked in the maps with the same colouring. (e) Integrated intensity map over the high energy region [840, 940] nm marked in blue. (f) Integrated intensity map over the low energy region [940, 1070] nm marked in red. The scale bars in (b, c, e and f) are 2 μm.

Room temperature PL spectra of Zn_3_P_2_ films grown between 180 °C and 250 °C and V/II ∼ 1 are presented in Fig. 4a. For all growth conditions, the spectra resemble the previously reported room temperature PL emission of Zn_3_P_2_, characterised by a direct bandgap emission around 810 nm (∼1.53 eV) and a defect related peak around 875 nm (∼1.41 eV).^10,11,22,26^

The origin of the defect peak is still debated. Different hypotheses proposed to explain this peak such as indirect band gap or indium contamination were disproven.^11,67,68^ A recent first principle study by Yuan *et al.*^16^ has shown that zinc vacancies (V_Zn_), situated at transition levels ∼0.14–0.2 eV above the valence band maximum (VBM), possess a very low formation energy and are likely to explain electrical properties and luminescence data reported in the literature. The energy position of these vacancies is consistent with the defect emission shown in Fig. 4a. For the sake of clarity, those simulation conditions are quite different from the experimental setup – namely stoichiometric compound at 0 K with isolated point defects. For example, Stutz *et al.*^63^ have measured off-stoichiometric Zn_3_P_2_ thin films and estimated based on an increased crystal lattice that the defect concentration would be in the order of 10^20^ to 10^21^ cm^−3^, if made up exclusively by phosphorus interstitials (P_i_).

In addition to intrinsic defects, Spadaro *et al.*^25^ have found that an interface between rotated crystallites in Zn_3_P_2_ can appear. These interfaces create shallow states around 0.1 eV, 0.21 eV and 0.38 eV, matching well with the experimentally measured emission. Similar rotated domains along the (101) plane were found in the presented q-vdW epitaxially grown Zn_3_P_2_. TEM images are provided in Fig. S16 in the SI. While the interface at the (101) plane does not form shallow states, a shrinkage of the band gap was reported. The finding of the rotated (101) interface also indicates the likely existence of other rotated interfaces, which are accompanied with shallow states.

On the basis of these reports, it is likely that the emission peak at 875 nm here observed includes contributions from both V_Zn_ point defects and extended defects such as grain boundaries.

PL spectra in Fig. 4a exhibit a significant decrease in emission intensity for lower growth temperatures. STEM-EDX measurements in Fig. S4 in the SI indicate similar thickness values for all the characterised films. The decrease in luminescence thus highlights a variation in the quality of the film as a function of the growth temperature. The same trend is observed in cryo-CL measurements, given in Fig. S15 in the SI. The PL and CL emission point out that the grain boundaries do have a lesser impact on the room temperature emission properties compared to growth condition variations of the polycrystalline thin films. Fluctuations in RT-CL emission (Fig. S17 in SI) can be observed but are suspected to be related to the variation in thickness of the thin film. The simulations of Yuan *et al.*^16^ provide a good explanation for the decrease in emission intensity. They found that all intrinsic defects except V_Zn_ give rise to deep trap levels. We assume that the increased P content in the films leads to a higher concentration of P_i_ and P_Zn_ in these films. In return, this can lead to a higher density of non-radiative recombination centers. XRD rocking-curve measurements also indicated an improvement of the crystal quality with increasing growth temperature, which might be linked to a reduction of extended defects at grain boundaries. In general, we found that higher growth temperatures lead to thin films with better quality for solar energy conversion.

To date, CL measurements have not been reported for Zn_3_P_2_ thin films due to extremely weak or even non-existent CL emission. While the tendency of delamination of q-vdW grown Zn_3_P_2_ films complicates device fabrication, this separation from the substrate enhances luminescence. The stronger luminescence can be observed both in CL and PL and is especially pronounced in sub-bandgap emission. Comparative CL and PL maps of delaminated films and those in contact with the substrate are provided in Fig. S17 in the SI. The CL measurements in this study focus on delaminated flakes rather than substrate-bound films. Fig. S13 in the SI also shows the comparison of room temperature PL spectra of Zn_3_P_2_ films transferred from graphene to Si_3_N_4_ and Au. While the PL emission intensity of Zn_3_P_2_ on Au is very similar to the one on graphene, emission is enhanced when the thin film is in contact with a dielectric material. Enhanced emission of free-standing films may therefore be due to the hindrance of diffusion and recombination or extraction of carriers in the graphene.

Fig. 4b–f show the SEM image and the corresponding CL maps derived from the hyperspectral data taken at 10 K. The measured thin film is grown on a Si-face H-CVD substrate for 6 h at 230 °C with a V/II flux ratio of 1. The CL emission is in accordance with low temperature PL measurements of Zn_3_P_2_ grown on wet-transferred graphene reported by Paul *et al.*^26^ and monocrystalline Zn_3_P_2_ on InP reported by Stutz *et al.*^10^ At 10 K, the bandgap emission is quenched, and two distinct emission regions below the bandgap are present. We refer to the first one as high energy emission, positioned around 900 nm (∼1.38 eV) and the second one as low energy emission, situated around 980 nm (∼1.27 eV). Fig. 4d highlights exemplary spectra corresponding to the same regions marked in the CL maps. From these spectra, we can observe that the high energy emission is composed of only one peak, while the low energy emission contains several emission peaks depending on the location on the thin film.

Spatial variation in emission intensity can be observed in the panchromatic map in Fig. 4c. A comparison between the panchromatic map and the topography of the thin film, shown in the SEM image, indicates a potential correlation. A further division of the spectrum into the high energy and low energy emission is given in Fig. 4e and f, showing the integrated intensity maps over the two marked regions in Fig. 4d. From these maps we can see that the high energy emission is present throughout the entire sample and the emission intensity shows some correlation with the surface topography. Stronger emission, up to a factor of 2, is observed for rough topography regions in the SEM image. We speculate that this variation in emission intensity is unrelated to changes in the material itself as STEM-EDS did not reveal significant changes within the thin film. We also don't expect thickness variations to be the cause of the stronger emission, as the penetration depth of the electron beam is below the film thickness. Casino simulations of the penetration depth are given in Fig. S18 in the SI. The correlation between the topography and the high energy emission is still under investigation. Paul *et al.*^26^ showed that this high energy emission blue-shifts to 875 nm at RT and therefore has the same origin as the defect emission observed in RT-PL as previously discussed.

More striking is the spatial and intensity variation of the low energy emission. It appears strongly localised and, when present, much more intense than the high energy emission peak. This is even more surprising when considering that the CCD used in the CL setup has a strong decrease in the efficiency for wavelengths above 900 nm. The emission doesn't show a clear correlation to the surface topography. The low energy emission was reported to appear only at temperatures below 150 K and its origin is still debated. Stutz *et al.*^10^ mentioned the possibility of the low energy emission being related to band tail recombination mechanisms, which could originate from high concentrations of charged defects as present in off-stoichiometric Zn_3_P_2_. Yuan *et al.*^16^ on the other hand found that these low energy emissions match well with the transition levels of P_Zn_ and Zn_P_ (∼0.35–0.45 eV).

Based on the result of our study, we also add grain boundaries or strain at the grain boundaries, discussed in section 3.3, as a potential cause of these localised low energy emissions. Preliminary DFT simulations show that strain can have a significant influence on the band structure of Zn_3_P_2_. Further investigations on the effect of strain on the emission of Zn_3_P_2_ are ongoing and will be presented in a separate study.

To summarise, we ascertain that luminescence of Zn_3_P_2_ films is brighter for thin films grown at higher temperatures, which contain more Zn. CL measurements demonstrate that the luminescence response of the thin film is not homogeneous at the μm scale. In particular, we detected very strong and spatially localised emissions at a photon energy below the bandgap, which may be related to grain boundaries or strain. The origin of the emission peaks within the bandgap of Zn_3_P_2_ is however still debated, and further studies are needed to conclude with certainty.

## 4 Conclusion

The growth of Zn_3_P_2_ on five different graphene substrates was studied. We found that topographical features such as steps and wrinkles act as main nucleation sites as long as the graphene is of high quality. If the graphene is unstable during the growth process or generally very defective, these defects become the main nucleation sites. The ideal substrate for Zn_3_P_2_ thin film growth according to our observations would be the Si-face H-CVD substrate due to the high quality, large graphene coverage and homogeneous topography.

Our results reveal that despite the different characteristics of the tested substrates, the growth window of crystalline Zn_3_P_2_ is the same on all graphene substrates, meaning that the composition and grain morphology are affected only by the growth conditions, but not by the cause of the nucleation. A continuous change in composition was observed when changing growth conditions, similar to previously reported Zn_3_P_2_ growth on other substrates. The compositional variation is also accompanied by a continuous change in grain morphology from round to triangular. Our results showed that the growth of Zn_3_P_2_ on graphene is inherently polycrystalline, regardless of the growth conditions or graphene substrate choice.

XRD measurements and optical emission properties of films of different growth conditions pointed towards an increase in quality when growing at higher temperatures. We conclude that high temperature growth is more desirable for thin films used in future solar cell devices. Cryo-CL measurements revealed the presence of an extremely bright low energy emission peak localised in micrometric spots. We assume the grain boundaries and the observed localised strain to contribute to the low energy emission. Additional studies are needed to confirm this hypothesis.

While the growth on graphene substrates offers the advantage of replacing rare substrate materials and enabling exfoliation, new challenges remain. Grain boundaries and strain within the polycrystalline thin film can significantly affect its optical and electrical properties. Additionally, delamination was found to limit the thickness and size of crack-free thin films, which makes the choice of substrate an important factor for q-vdW grown Zn_3_P_2_. Future work will determine whether the benefits of having more freedom in the device design outweighs the drawback of having grain boundaries, and whether quasi-van der Waals epitaxy of Zn_3_P_2_ can compete with other growth techniques or materials in solar energy applications.

## Author contributions

TH carried out the epitaxial growth of Zn_3_P_2_, AFM, XRD and optical characterisation of Zn_3_P_2_ and graphene as well as the writing process. HRF undertook the STEM characterisation, including STEM-EDS and GPA analysis. CM, AEA and AM produced and provided the Si-face H-CVD substrates. SM contributed to the data analysis for the CL measurements. NK worked on the DFT simulations, supervised by SB. RL, KAW and DD contributed to the design and realization of the epitaxial growth. JA and MCS supervised the STEM characterisation. VP revised the manuscript and supervised the writing process. AFM outlined the research project, directed the work and contributed to the writing of the manuscript.

## Conflicts of interest

There are no conflicts to declare.

## Supplementary Material

CE-028-D5CE00351B-s001

## Data Availability

The raw data have been uploaded to https://doi.org/10.5281/zenodo.17358926 and https://doi.org/10.34810/data2691. Supplementary information is available. See DOI: https://doi.org/10.1039/d5ce00351b.
