# Supplementary material for: Link between graphene features and the resulting functionality of quasi-van der Waals Zn3P2
Source: CrystEngComm. 2025 Oct 29;28(3):635–48. doi: 10.1039/d5ce00351b (PMC12621204; doi:10.1039/d5ce00351b)
Supplement: CE-028-D5CE00351B-s001 [file CE-028-D5CE00351B-s001.pdf]

Supplementary Information (SI) for CrystEngComm.
This journal is © The Royal Society of Chemistry 2025

Supporting Information for: Link between graphene features and the resulting functionality of quasi-van der Waals Zn_3P_2

*

E-mail: anna.fontcuberta-morral@epfl.ch

Thomas Hagger,[1] Helena Rabelo Freitas,[2] Chiara Mastropasqua,[3] Ahmed El Alouani,[3] Stefano Marinoni,[1] Nico Kawashima,[4,5] Raphael Lemerle,[1] Kamil Artur Wodzislawski,[1] Didem Dede,[1] Silvana Botti,[4,5] Maria Chiara Spadaro,[2,6] Valerio Piazza,[1] Adrien Michon,[3] Jordi Arbiol,[2,7] and Anna Fontcuberta i Morral[1]

[1]Laboratory of Semiconductor Materials, Institute of Materials, School of Engineering, Ecole Polytechnique Fédérale de Lausanne, 1015, Lausanne, Switzerland
E-mail: anna.fontcuberta-morral@epfl.ch
[2]Catalan Institute of Nanoscience and Nanotechnology (ICN2), CSIC and BIST, Campus UAB, Bellaterra, Barcelona, Catalonia, 08193, Spain
[3]Université Côte d'Azur, CNRS, CRHEA, Rue Bernard Grégory, 06560 Valbonne, France
[4]Research Center Future Energy Materials and Systems of the University Alliance Ruhr and ICAMS, Ruhr University Bochum, Universitätsstraße 150, D-44801, Bochum, Germany
[5]Institut für Festkörpertheorie und-optik, Friedrich-Schiller-Universität Jena, Max-Wien-Platz 1, 07743 Jena, Germany
[6]Department of Physics and Astronomy "Ettore Majorana", University of Catania, via S. Sofia 64, 95123 Catania, Italy - CNR-IMM, via S. Sofia 64, 95123 Catania, Italy
[7]ICREA, Pg. Lluís Companys 23, 08010 Barcelona, Catalonia, Spain

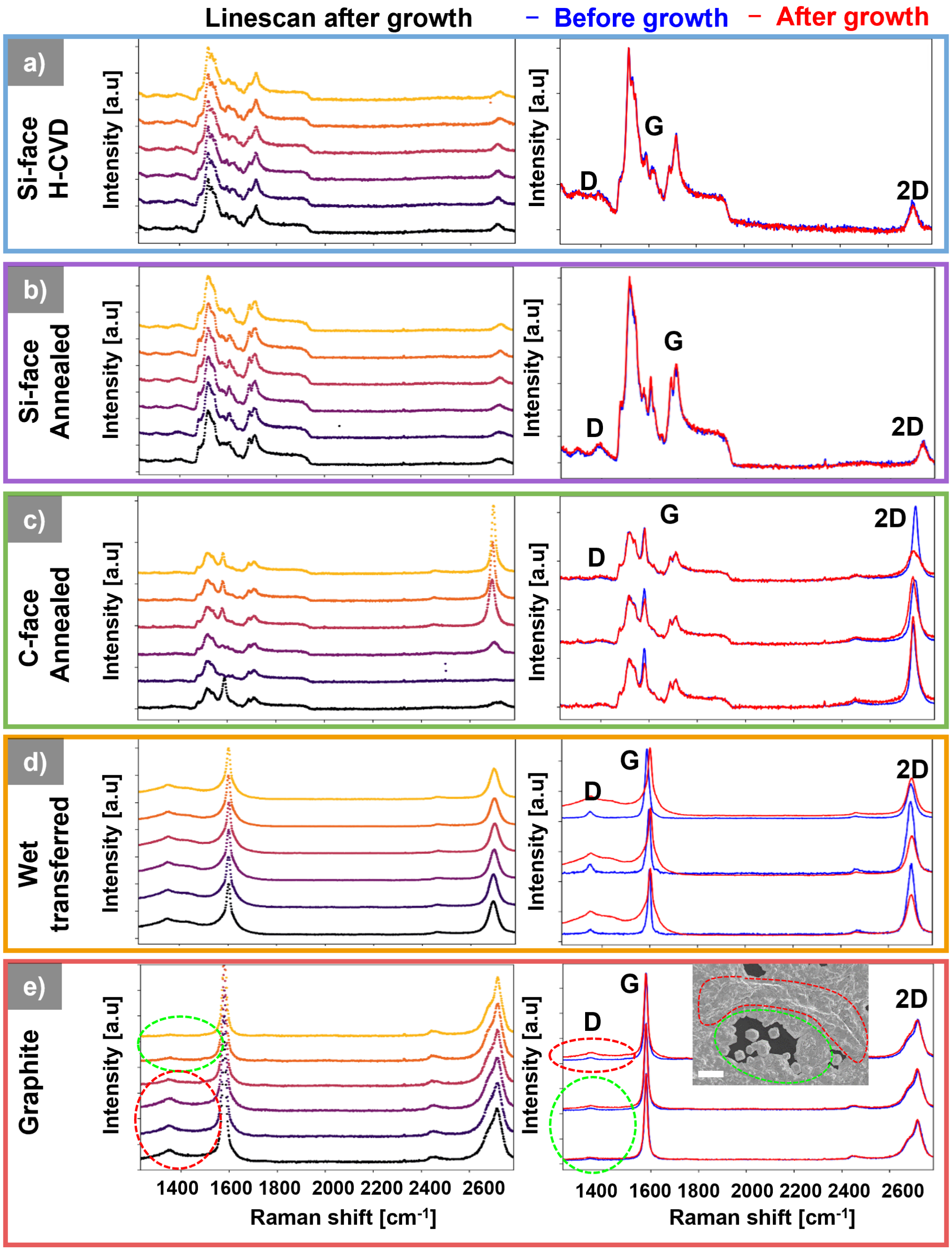

Figure S1: Raman spectra of the five graphene substrates without the subtraction of the SiC substrate for (b-d). Left column shows line scans of the substrates after a growth process. Line scans were taken with 500 nm steps between each spectrum. The right column shows spectra before (blue) and after (red) growth. Exemplary spectra were chosen to indicate differences before and after. Graphite substrates have an SEM inlet indicating growth on non-defective/stable (green) graphene and more defective/ unstable graphene (red) leading to a worse quality film. Scale bar of the SEM image is 2 μm. The Raman spectra are not taken at the same positions as the SEM image.

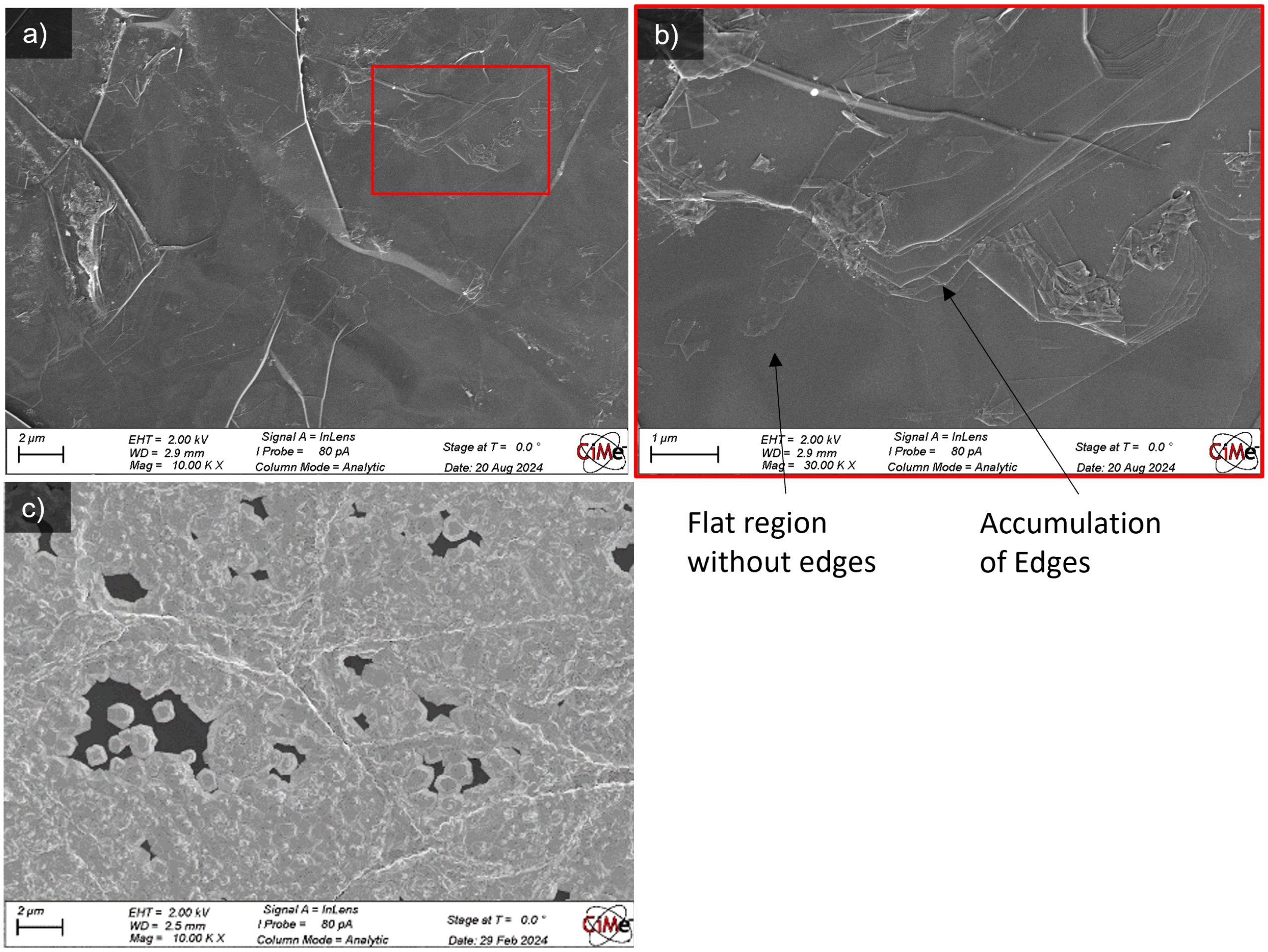

Figure S2: (a-b) SEM images of graphite before growth and (c) after growth. The before and after growth images were taken on different positions on the substrate. One can observe, that the growth on flat regions leads to bigger grains due to a smaller nucleation density while the growth on wrinkles and accumulated edges of graphene layers lead to a higher nucleation density.

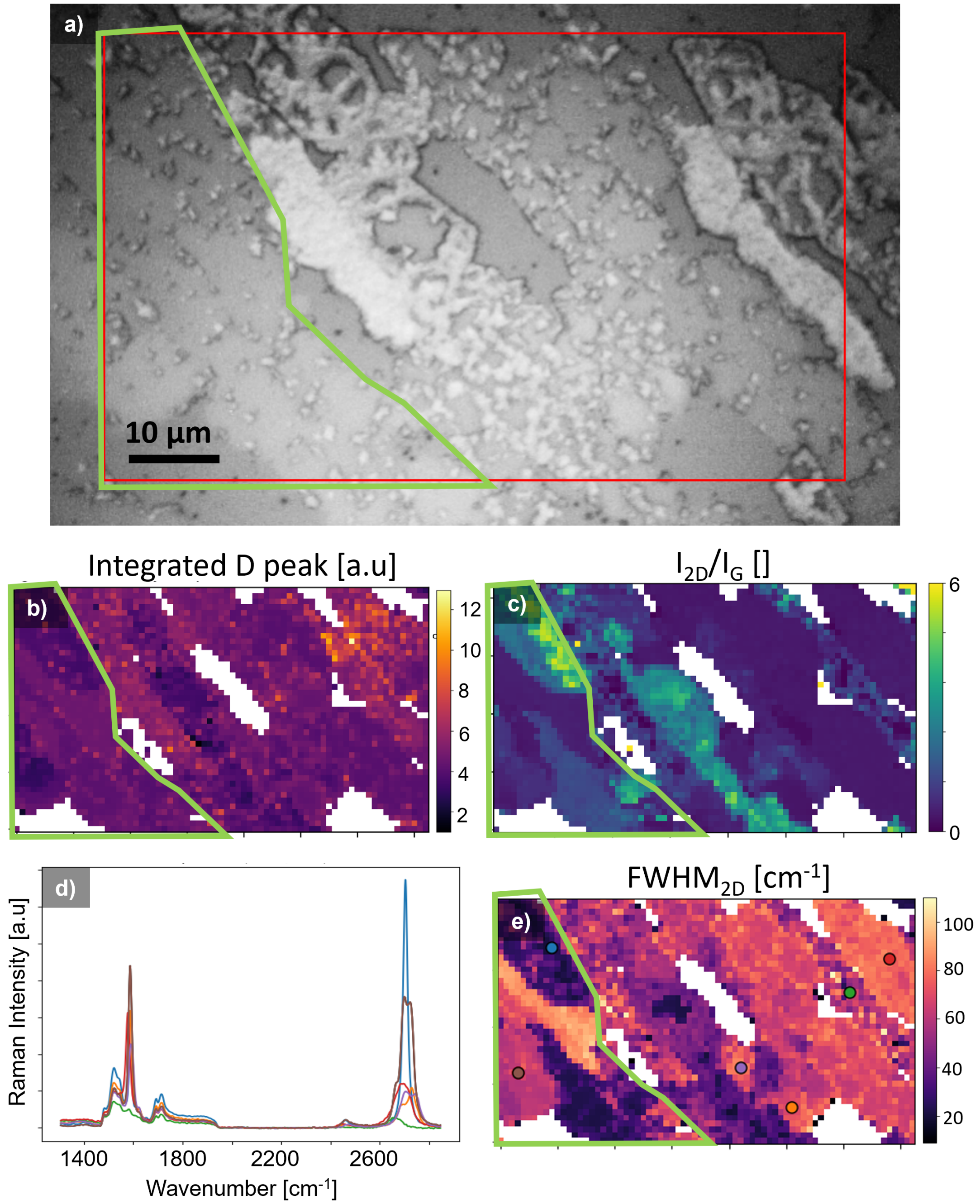

Figure S3: Raman mapping of the graphene on C-face annealed SiC substrate after the growth of Zn_3P_2. The Measurement was conducted with a 532 nm laser and (a) shows the microscopy image of the mapped area. Lighter contrast is coming from the Zn_3P_2. (b) shows the integrated area in the range of the D peak [1250-1450] cm^{-1} without the removal of the SiC background. (c) shows the intensity ratio of the 2D vs G peak. (d) shows some exemplary spectra of different regions that are marked in (e), the map of the $FWHM_{2D}$.

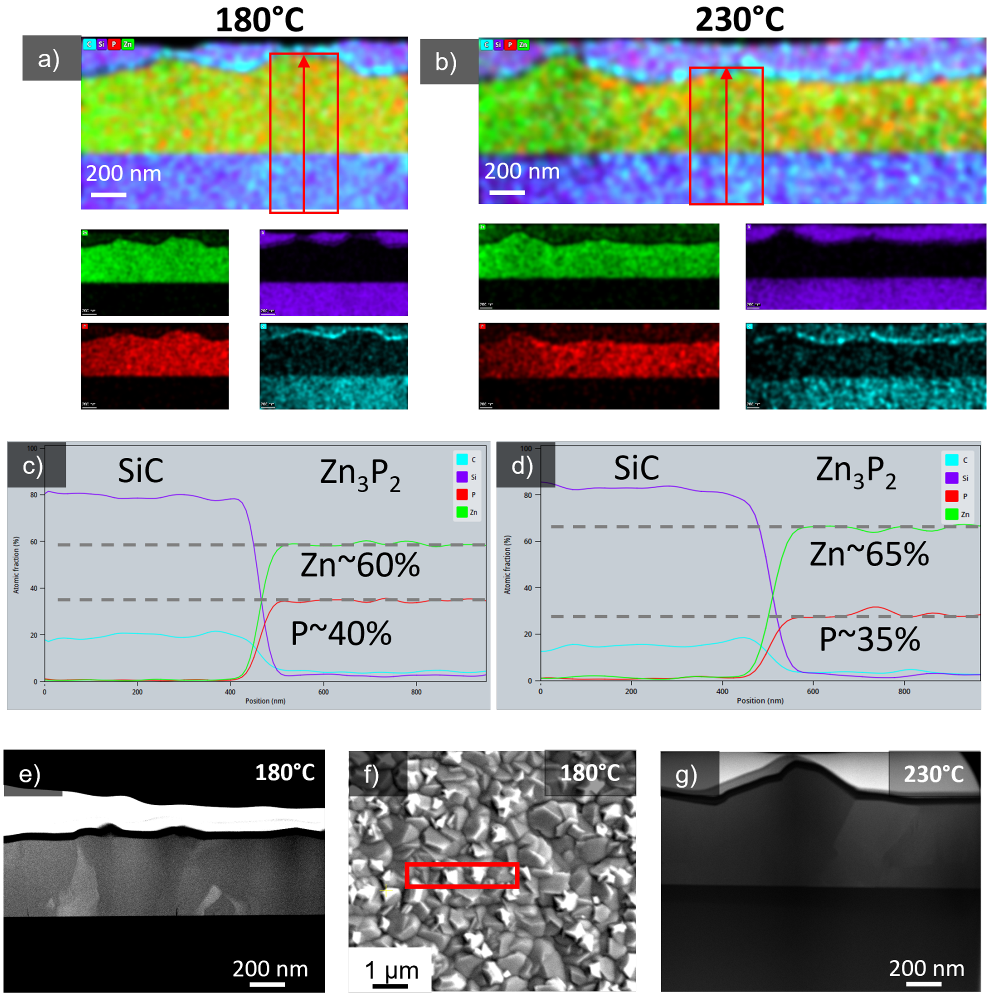

Figure S4: (a) STEM-EDX elemental mapping measurements of a film grown at 180°C and a V/II=1 indicating a composition close to stoichiometry; (b) STEM-EDX measurements of a film grown at 230°C and a V/II=1, with a higher Zn concentration detected. (c-d) show a linescan over the thin film for each growth condition. (e) shows the high angle annular dark field (HAADF) STEM image for the 180°C growth, and (f) the top view SEM image with indicated location where the lamella was cut. (g) shows the HAADF TEM image of the 230°C growth. HAADF STEM images do not show the precise location where the EDX map was acquired but clearly indicate the presence of grain boundaries throughout the thin films.

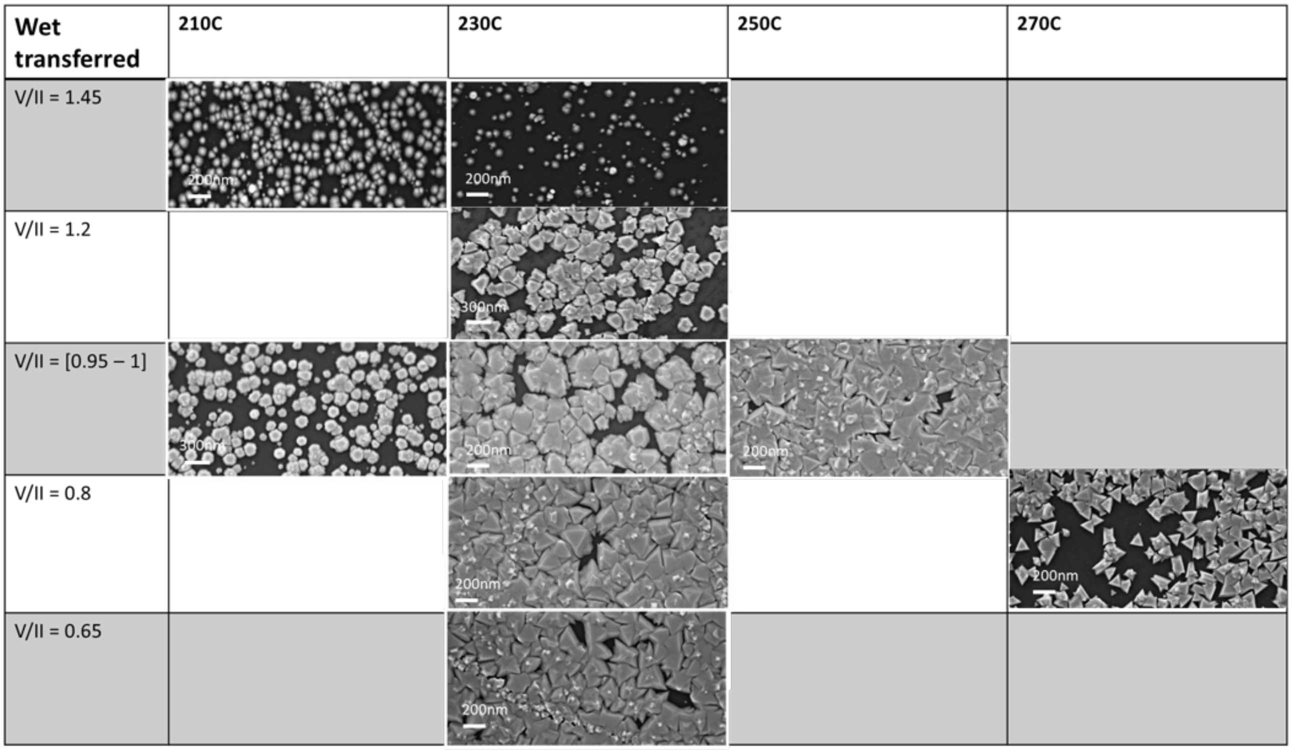

Figure S5: SEM top view images of the growth window on wet-transferred graphene from Graphenea. The grains follow the same trend in morphology as reported in the main text. The growth temperature is increased by around 30°C due to the different heat absorption compared to the SiC substrates. Scale bars of the images are 200 nm.

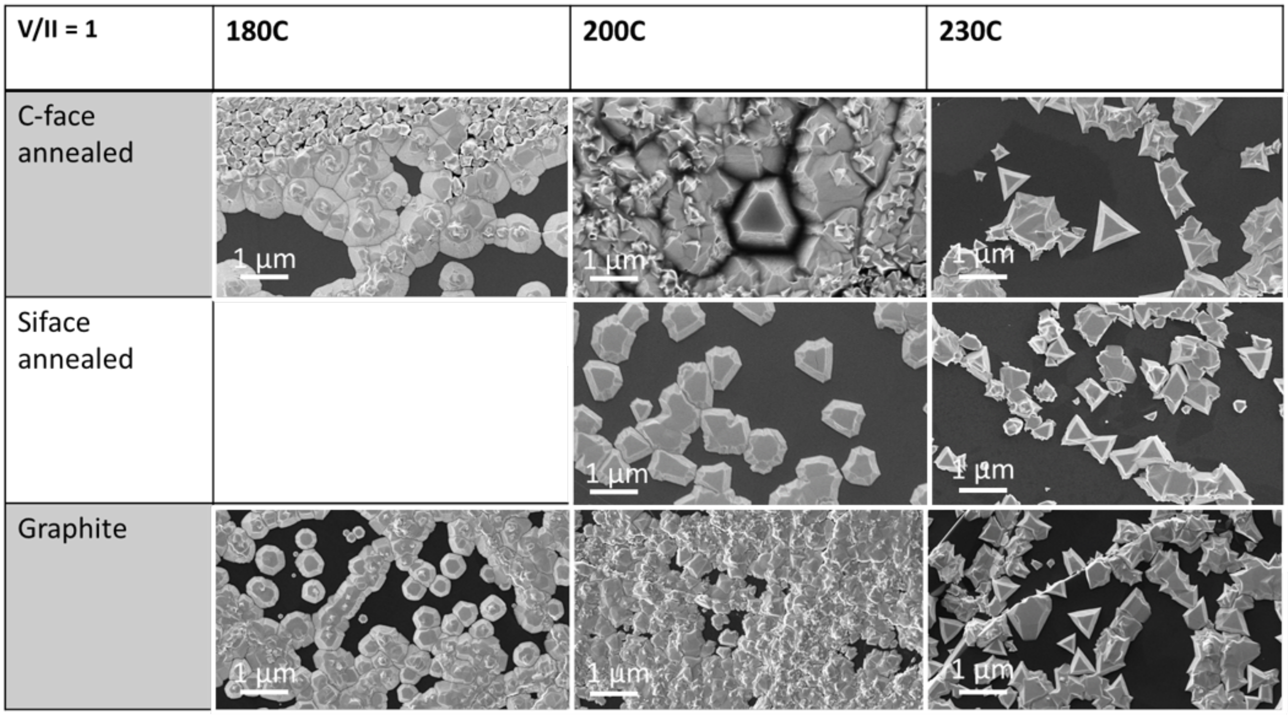

Figure S6: SEM top view images of the growth window on C-face, Si-face annealed and graphite substrates. Here all samples were grown at a V/II=1. The grains follow the same trend in morphology as reported in the main text. Scale bars of the images are 1 μm.

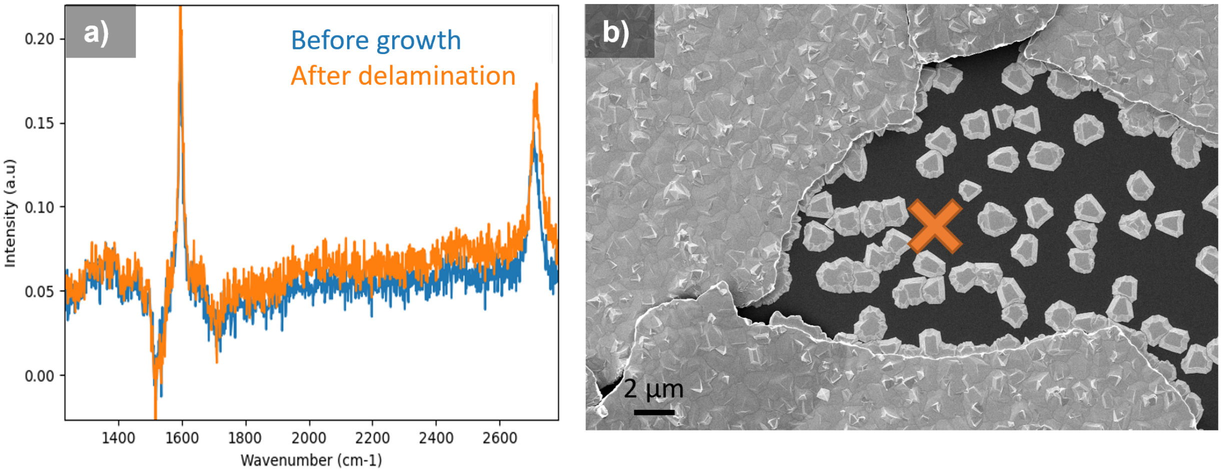

Figure S7: (a) Raman spectra of the five graphene substrates with the subtraction of the SiC substrate signal. The after growth measurement was taken on a delaminated part, showing the non-destructive process of delamination similar to the indicated position in SEM image in (b). SEM image also shows the same quality of growth as for the initial growth stage. Growth conditions were 200°C growth temperature and V/II flux ratio = 1.

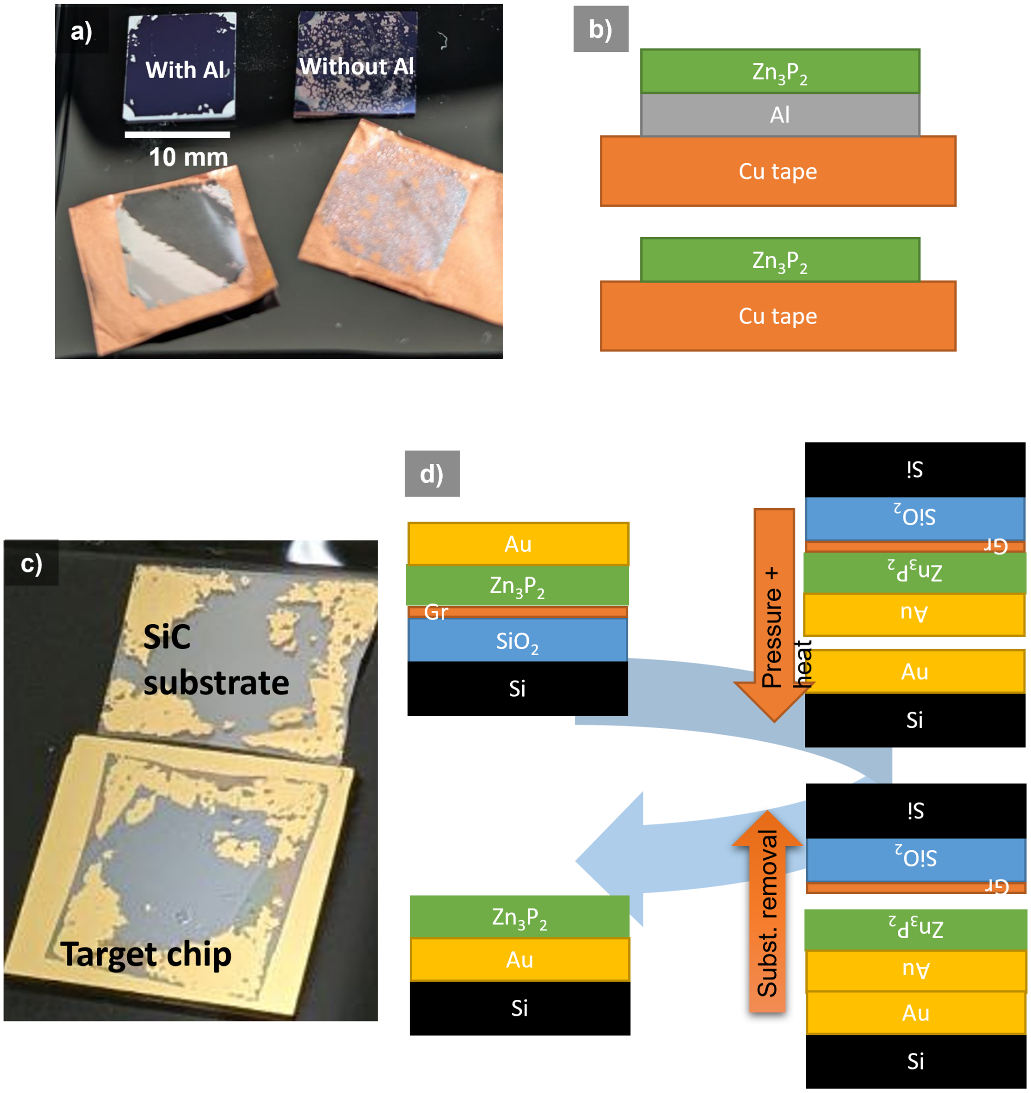

Figure S8: Two exemplary transfer processes of Zn3P2. (a) shows the transfer on a copper tape, with and without the use of an Al layer deposited on Zn3P2 before the transfer. Schematic is given in (b). The Al layer provides additional stability to the 150 nm thick Zn_3P_2 film and acts as a stressor layer which improves the separation from the graphene interface. (c) shows the outcome of the thin film (grey) transferred on a 1x1 cm^2 gold covered Si chip after thermo-compression bonding. In this process, the gold surfaces are first activated with an Ar plasma and then directly heated up to 150°C and pressed together at 0.5 MPa, fusing the gold surfaces together. (d) shows the process flow of the transfer. Substrate removal does not usually require additional force since the two sides have already separated once the pressure is released,

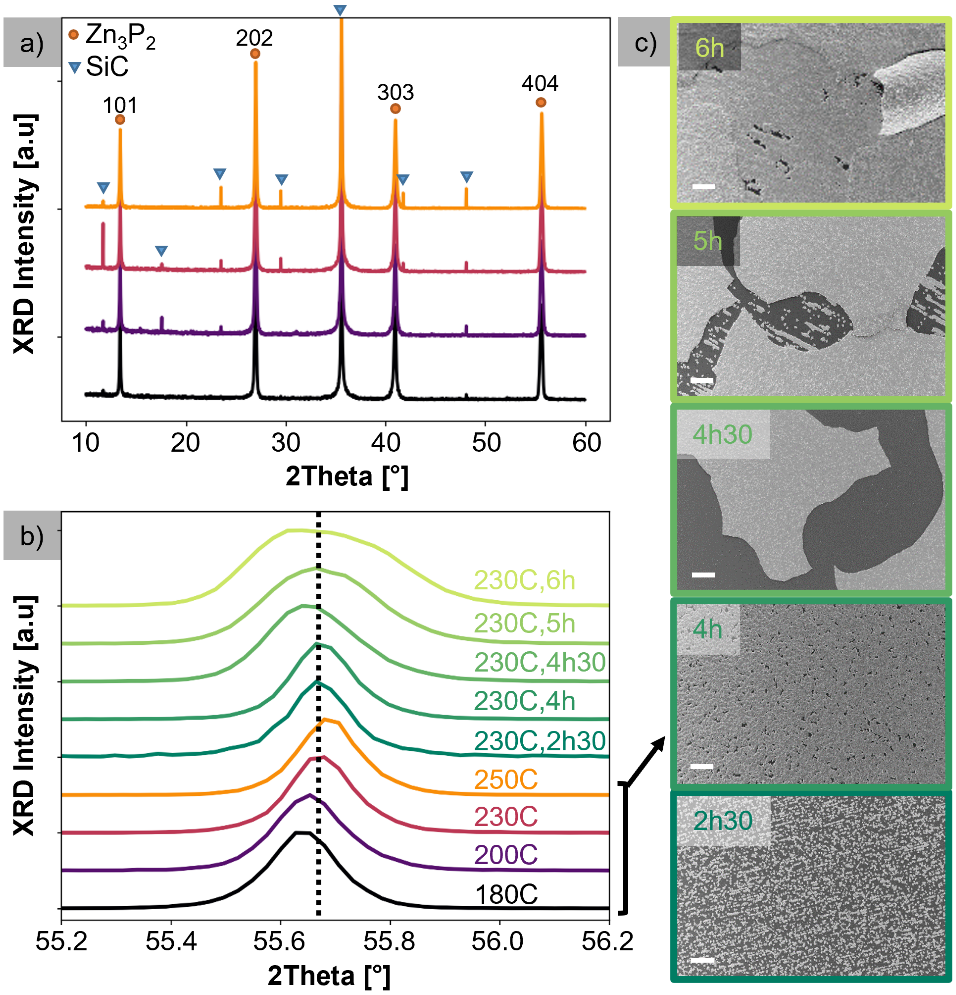

Figure S9: (a) Zn_3P_2 in an XRD Bragg-Brentano ($\theta-\theta$) configuration grown at four different growth temperatures with similar V/II flux ratios, showing that all growth conditions lead to highly textured Zn_3P_2 films along the (101) direction; (b) shows the 404 peak of Zn_3P_2 grown at four different growth temperatures with similar V/II flux ratios and five samples grown for different duration at 230°C. The samples grown at different temperatures all consist of films that have a coverage similar to the 4h growth marked in (c); (c) Shows the SEM top view images of the five samples grown for different duration at 230°C. Scale bars of the images are 10 μm. One can see a peak shift as well as broadening and asymmetry related to delamination of the film.

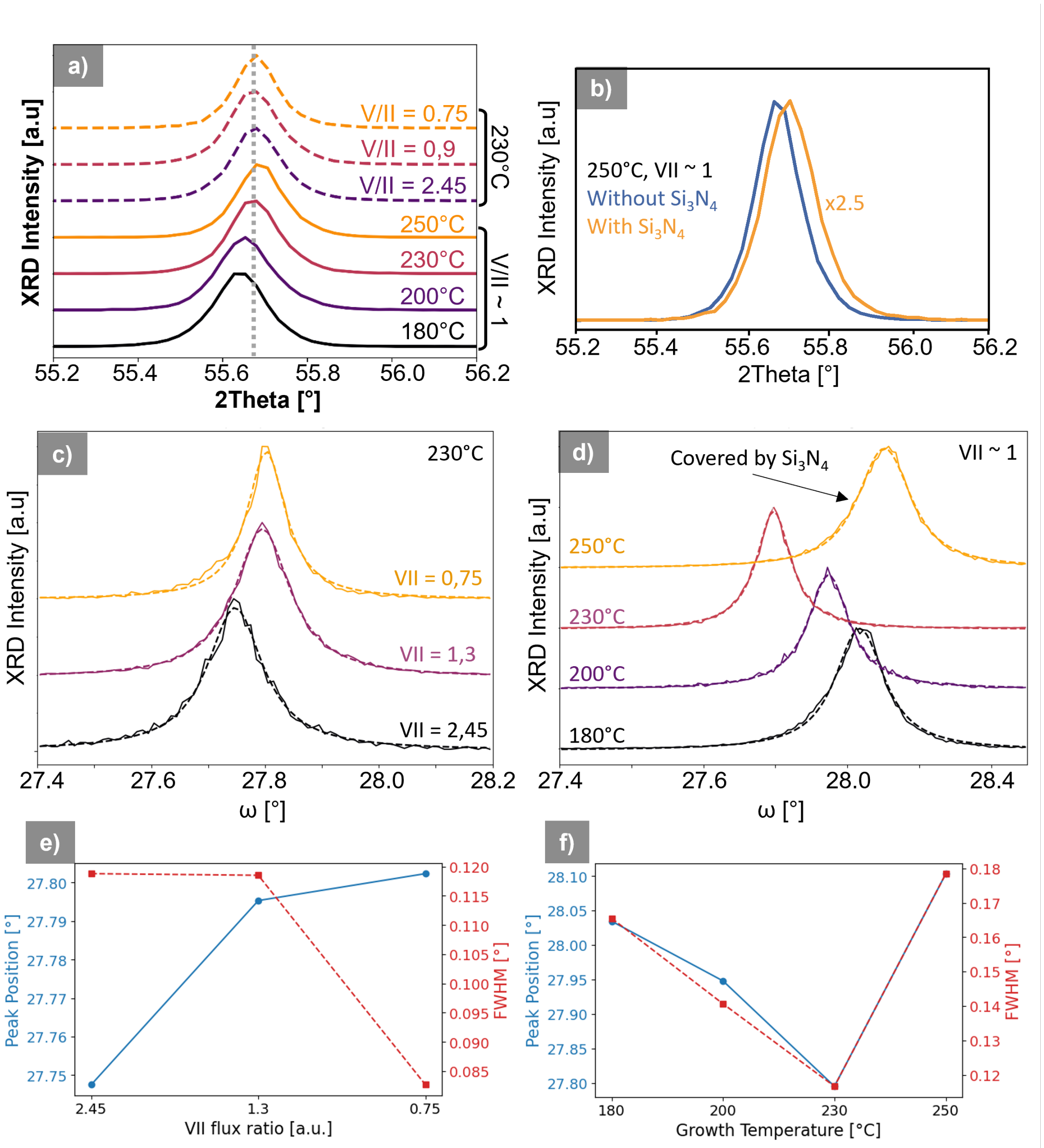

Figure S10: (a) Brag-Brentano scan of different growth conditions (b) Comparison of the (404) peak of the film grown at 250°C and V/II ≈ 1 without and with 200 nm Si_3N_4. The covering of Si_3N_4 or oxidation of the film (measured only around 1 year after growth) prior to the covering might be the cause of broadening and shifting of the peak. (c) Zn_3P_2 in an XRD rocking-curve (ω-scan) configuration grown at three different V/II fluxes at growth temperature of 230°C and (d) of four different growth temperatures at V/II ≈ 1. To be noted, that the rocking-curve of the film grown at 250°C and V/II ≈ 1 has only been measured once covered by 200 nm Si_3N_4. Panel (e) and (f) shows the peak position and FWHM of a Lorentzian fit of the rocking curves in (a) respectively (d). Rocking curve measurements have been centred at the specific position of the 2 position of the (404) peak of each sample. The narrowing of the FWHM when increasing the growth temperature could indicate higher quality and a smaller mosaic spread. It is unclear whether the peak position is an artifact from mounting the sample or related to general tilt of the lattice planes.

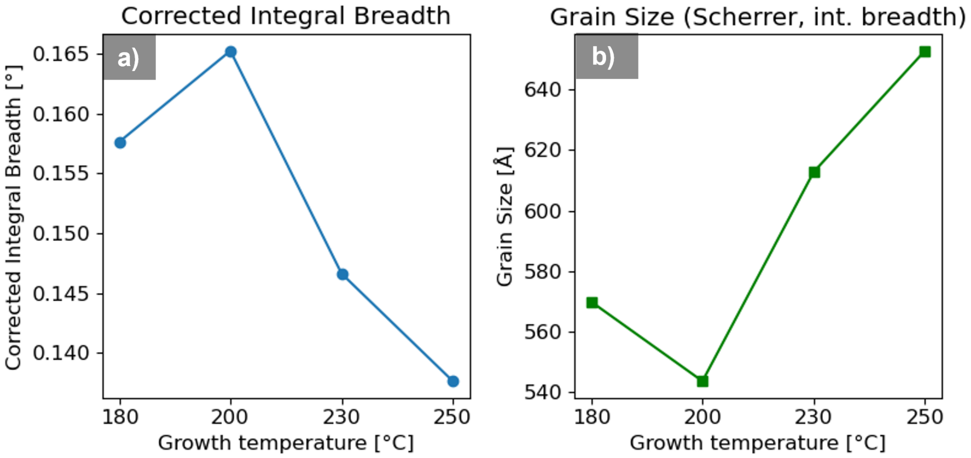

Figure S11: (a) Corrected integral breath of the XRD (404) peaks, corrected by the instrument broadening, measured with SRM 660c (Lab_6) for the four thin films of different growth temperatures shown in the main text in Figure 2c. (b) Calculated coherent domain size from Scherrer equation with the corrected integral breath along the growth direction (101). A trend of increasing coherent domain size is observed when increasing the growth temperature, indicating better crystallinity.

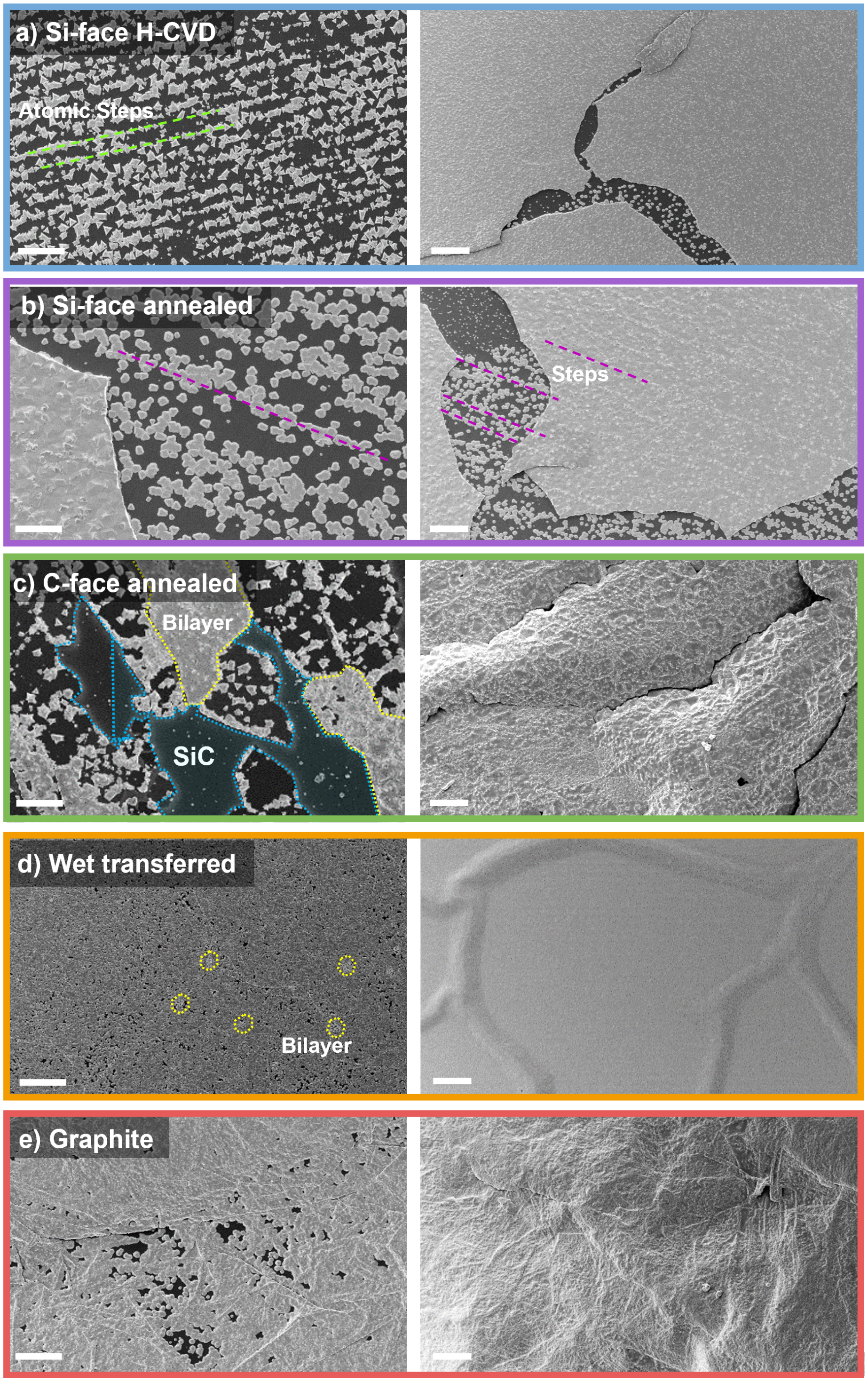

Figure S12: SEM top view images of Zn_3P_2 films grown on the five different graphene substrates. Scale bars are 5 μm in the left and 10 μm in the right column. Delamination of the film occurring for all films but the graphite likely due to covalent bonds formed at the edge of graphene flakes.

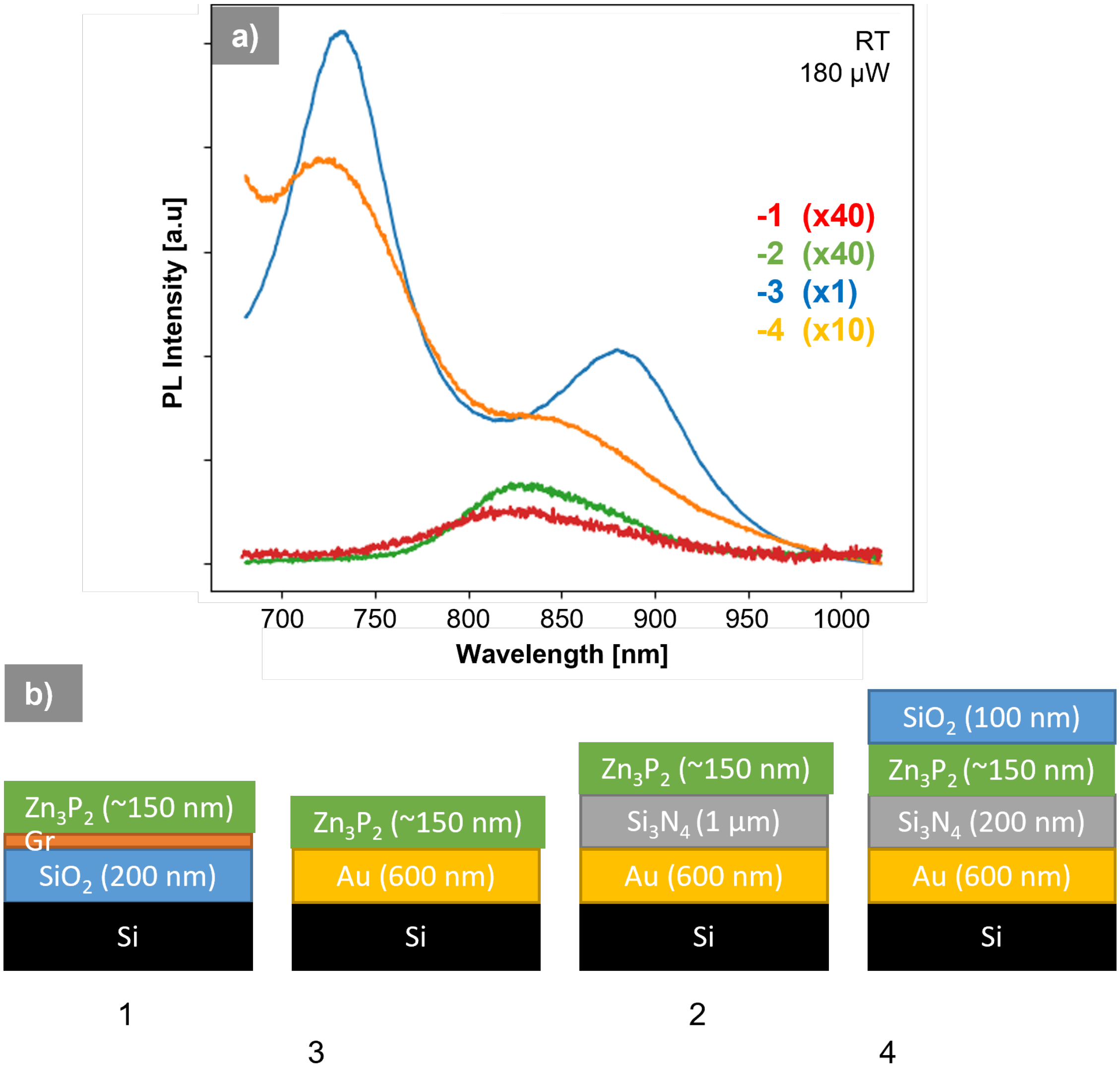

Figure S13: (a) Single point room-temperature PL spectra of four different configurations, represented in (b) Intensities of the spectra were scaled by the factor written in brackets in the legend. Configuration 2-4 have been obtained with the thermo-compression bonding. One can see, that Zn_3P_2 as grown and on gold have similar intensities. Adding a dielectric between the Zn3P2 and gold seems to increase the emission. It is still unclear why configuration 3 and 4 have such big differences in the emission of Zn_3P_2.

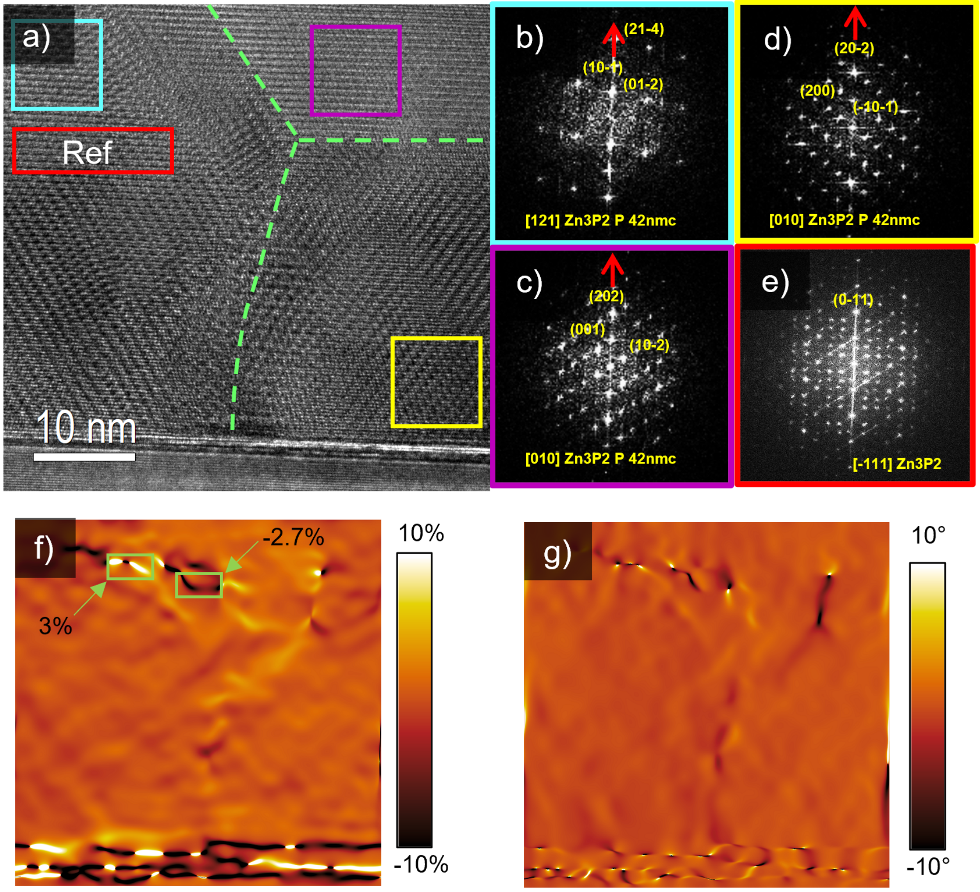

Figure S14: (a) Same HRTEM image of the multigrain Zn_3P_2 cross-section as shown in the Figure 3 in the main text; (b-e) indicating FFTs for the different grains; (f) shows dilatation and (g) rotation maps with respect to the Ref. in red.

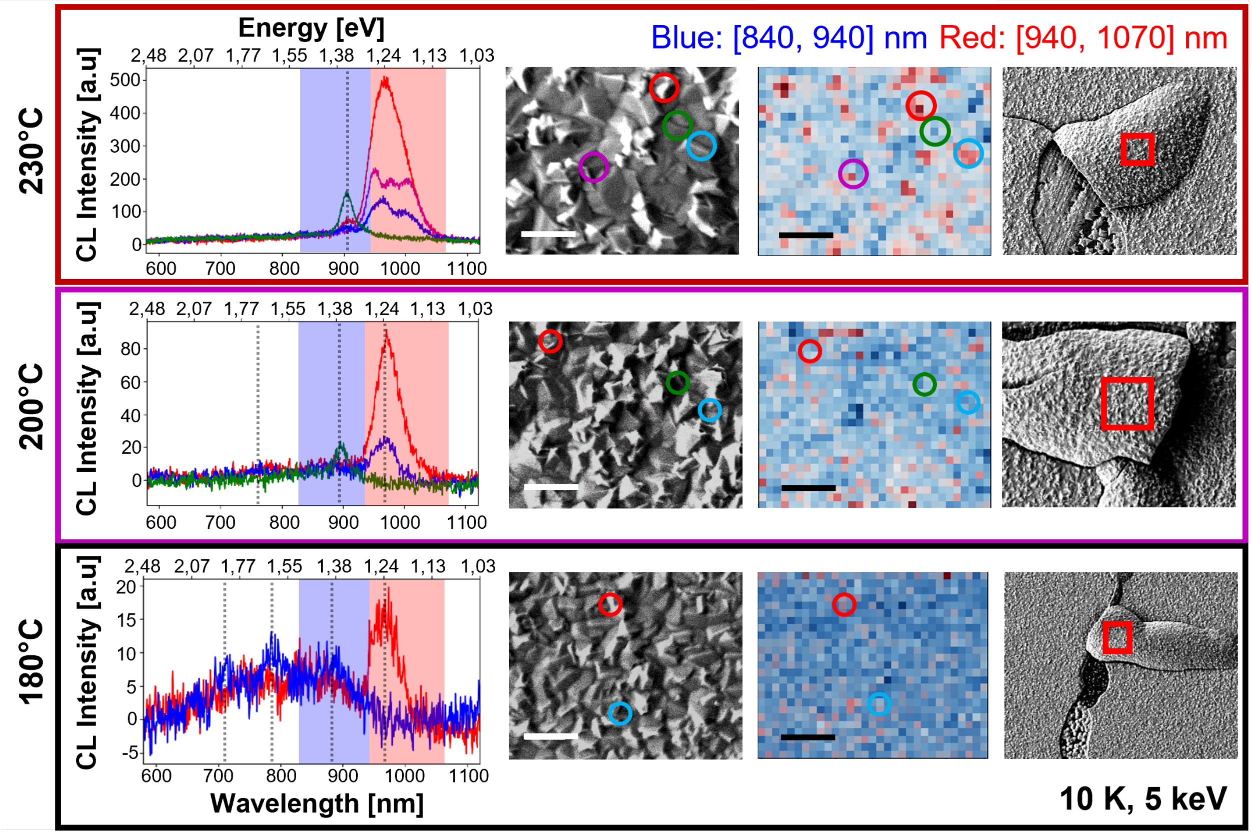

Figure S15: Comparison between CL spectral maps, taken at 10 K of Zn_3P_2 films grown at 230°C, 200°C and 180°C with a V/II flux ratio = 1. Film thicknesses measured by AFM are around 750 nm, 600 nm and 800 nm respectively. Dashed lines indicate potential peak positions. Although the peak below 900 nm could also be introduced by the data smoothening and no clear conclusion can be made on them. Scale bar of SEM image and normalised integrated intensity map in red and blue are 2 μm. Right column shows the peeled flake and indicates the location of acquired map with a red square.

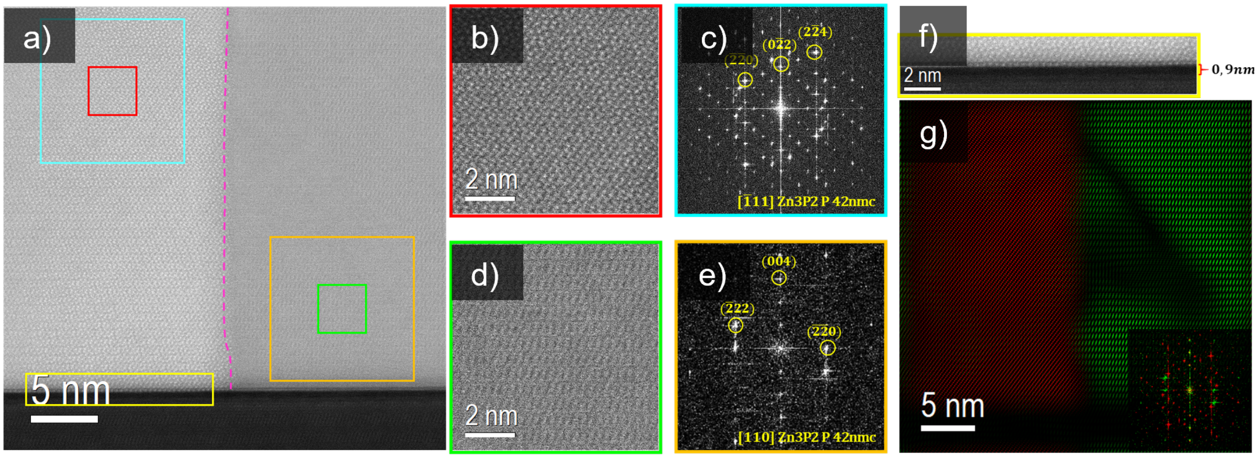

Figure S16: (a) STEM-HAADF image showcasing two different regions of the Zn3P2 film, probably two different domains/islands. The region on the left (b-c), highlighted in cyan, is displayed in its [-111] zone axis and the growth plane is the (0-11), while the region on the right, highlighted in orange (d-e), is displayed in its [110] zone axis, while its growth plane is the (001). Both regions are separated by a boundary highlighted by the dashed pink line. The 6H-SiC substrate is not oriented to the point of providing information on its zone axis and growth direction. (f) The double layer of graphene has a thickness of 9 Å, and it is highlighted in yellow. (g) The left and right domains are highlighted in the frequency filtered map in red and green, respectively.

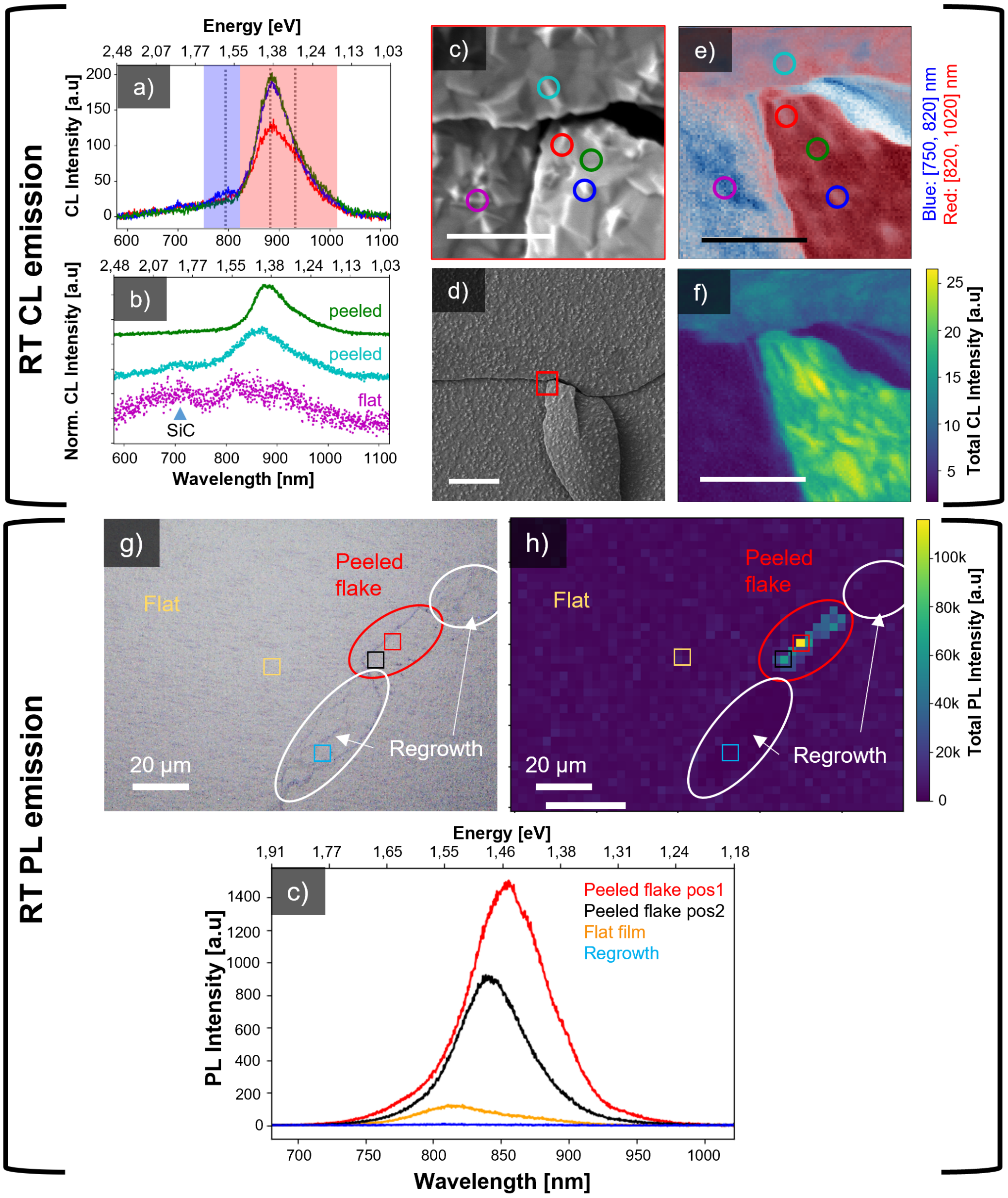

Figure S17: (a) Room temperature CL spectral maps of Zn_3P_2 films grown at 230°C with a V/II flux ratio = 1, acquired on peeled film; b) normalised spectra on differently peeled and flat (touching substrate) with positions indicated in (c), the SEM top view image of the map; (d) the zoomed out SEM image with scale bar of 10 μm and indicated region of the map; (e) the normalised integrated intensity map for the red and blue regions; (f) total integrated intensity map showing higher intensity for peeled film compared to adherent to substrate (flat) part. Scale bar of SEM image and normalised integrated intensity map in red and blue and total intensity map are 2 μm. Here the ranges in blue and red are not the same as for measurements taken at 10 K; (g) Microscopy image of the area of room temperature PL map; (h) Panchromatic PL intensity map indicating high PL emission intensity for peeled flakes; (i) Exemplary spectra selected at different positions in the map. Colours correspond to the marked positions in (g,h).

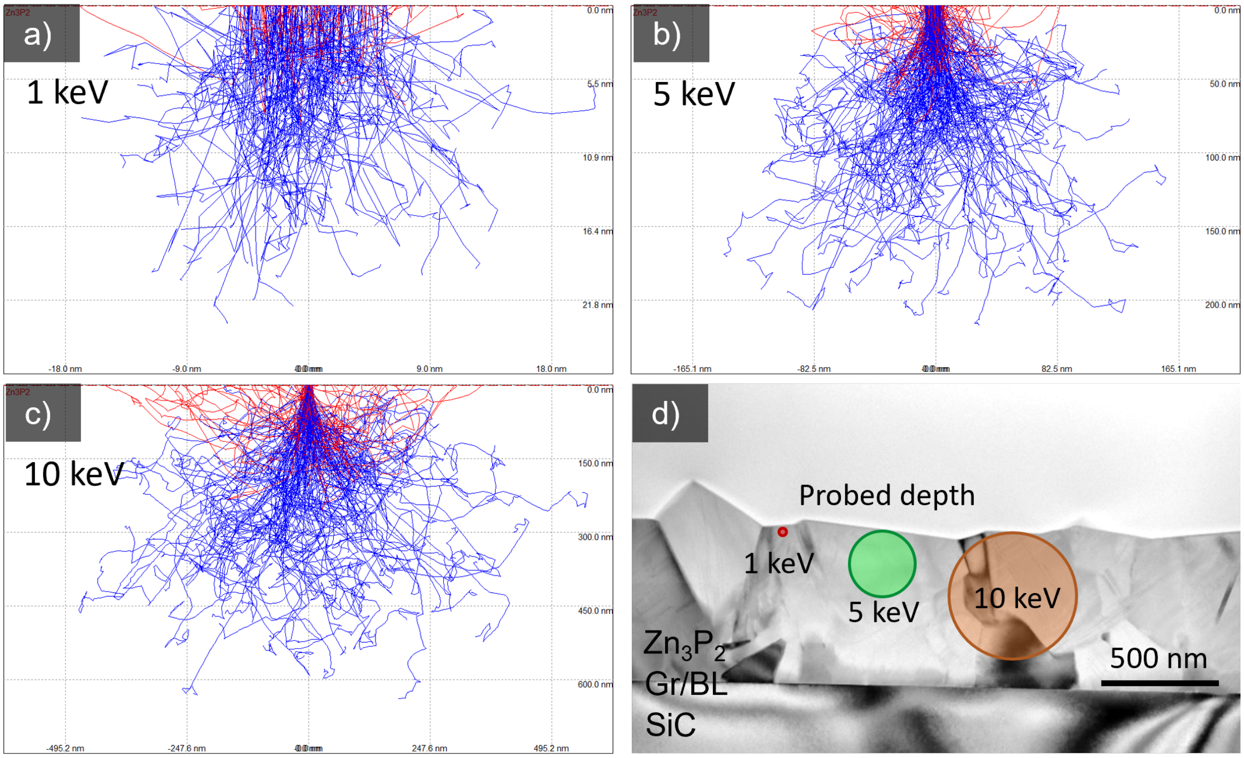

Figure S18: (a-c) Casino simulations, showing the interaction volume of Zn_3P_2 for different electron beam energies, 1, 5, 10 keV and (d) a comparison of this volume to the thickness of the film.
